# Multiple dimensions of reward-sensitivity and cognitive control relate to psychopathology: an exploratory structural equation modeling approach

**DOI:** 10.3389/fpsyg.2026.1823262

**Published:** 2026-09-11

**Authors:** Analicia K. Howard, Roselinde H. Kaiser, Naomi P. Friedman

**Affiliations:** 1 Institute for Behavioral Genetics, University of Colorado Boulder, Boulder, CO, United States; 2 Department of Psychology and Neuroscience, University of Colorado Boulder, Boulder, CO, United States; 3 Institute of Cognitive Science, University of Colorado Boulder, Boulder, CO, United States; 4 Renée Crown Wellness Institute, University of Colorado Boulder, Boulder, CO, United States

**Keywords:** anxiety, attention problems, depression, executive function, inhibitory control, sensation seeking

## Abstract

**Introduction:**

Abnormalities in reward sensitivity and cognitive control are observed in multiple psychiatric disorders. However, differences in measurement of reward sensitivity and cognitive control across studies have yielded mixed findings. Despite assumptions of convergence, the correlation between survey-based and task-based assessments of these constructs remains small.

**Methods:**

In this preregistered study of data from the Adolescent Brain Cognitive Development (ABCD) study (*N* = 11,875; ages 9–12 years), we examined relationships across these constructs and with psychopathology using exploratory structural equation modelling.

**Results:**

We found evidence for multiple cognitive control and reward sensitivity factors with modest correlations. Cognitive control and reward sensitivity independently related to both Internalizing and Externalizing psychopathology factors two years later (β = 0.01 to |0.605|).

**Discussion:**

Our findings highlight the utility of using multiple methods and raters to capture variance in reward sensitivity and cognitive control, showing they are multidimensional constructs each with unique relationships to psychopathology in adolescence.

## 1 Introduction

Understanding the individual differences related to psychopathology is paramount to identifying those at risk for developing associated disorders. Two constructs often implicated in psychopathology risk are cognitive control and reward sensitivity. Broadly defined, reward sensitivity is the propensity to identify, learn from, and gain gratification from positively valanced stimuli (Bornstein, 2018), whereas cognitive control is the ability to adaptively process information during goal-directed behavior (Badre, 2025; Miller and Cohen, 2001). Although both reward sensitivity and cognitive control are multidimensional (Berridge et al., 2009; Friedman and Miyake, 2017), they are often treated as unitary and measured with singular questionnaires or tasks, despite weak evidence for convergent validity across methods (Duckworth and Kern, 2011; Snyder et al., 2021). These constructs are often conflated with one another in the literature where subscales used to capture these constructs may contain aspects of both reward sensitivity and cognitive control (e.g., positive urgency in the Urgency, Premeditation, Perseverance, and Sensation Seeking scale; UPPS; Cyders and Smith, 2007). Given that both reward sensitivity and cognitive control are relevant to psychopathology (McCabe et al., 2021), understanding the underlying structure of items captured by common measures of these constructs is of particular interest. The Adolescent Brain and Cognitive Development (ABCD) study provides a unique opportunity to address this issue of construct validity by incorporating multiple tasks, surveys, and informants to examine how reward sensitivity and cognitive control jointly predict psychopathology in early adolescence.

### 1.1 Cognitive control and reward sensitivity: relations to psychopathology

The literature suggests reward and control constructs independently and jointly shape risk for adolescent psychopathology (Kaiser et al., 2023). Low cognitive control is related to both internalizing and externalizing disorders (Nigg, 2016; Ellingson et al., 2015; Pechtel et al., 2013; Harvey et al., 2005). Similarly, reward hypo- or hyper-sensitivity have been linked to diverse symptom dimensions (Baskin-Sommers and Foti, 2015; Alloy et al., 2012; Bjork et al., 2010). High reward sensitivity has been linked to substance use disorders, adolescent risk-taking, and mania (Dalley et al., 2011; Casey et al., 2008; Steinberg, 2008; Harden et al., 2017; Kaiser et al., 2023), whereas low reward sensitivity has been associated with anhedonic depression and social anxiety (Pizzagalli et al., 2005; Snyder et al., 2023).

Frameworks such as the National Institute of Mental Health's Research Domain Criteria (RDoC; National Institute of Mental Health, n.d.) address the dual influences of reward and control (Nielsen et al., 2019; Nigg, 2006). The RDoC seeks to link psychopathology to biologically grounded constructs, distinguishing reward responsiveness, and cognitive control across multiple units of analysis (Kozak and Cuthbert, 2016). Developmental neuroscience models (e.g., maturational imbalance, dual systems; Casey et al., 2008; Shulman et al., 2016; Steinberg, 2010) emphasize how early developing reward systems (peaking in mid-late adolescence) and later-maturing control systems (increasing through adulthood) contribute to adolescent risk-taking. Debate remains on whether reward sensitivity and control act additively or interactively: some models such as dual systems suggest an interaction (Nielsen et al., 2019; Willem et al., 2010), whereas accumulating evidence supports additive effects (Lozano Wun et al., 2025; Ellingson et al., 2019). Because we are primarily focused on investigating the structure of assessments of these constructs, rather than emphasizing how the reward and control interact across development, we focus on an additive model.

### 1.2 Measuring reward sensitivity and cognitive control

Cognitive control and reward sensitivity can be assessed with surveys or tasks. For example, the UPPS (Cyders and Smith, 2007; Whiteside and Lynam, 2003) survey includes subscales such as sensation seeking and premeditation, which are typically considered indicators of reward and control (Icenogle et al., 2017; Harden et al., 2017; Cyders et al., 2007; Cservenka et al., 2013). At the task level, cognitive control is measured with paradigms such as the flanker task, which assesses response inhibition and interference control, and reward sensitivity is assessed with tasks such as the delay discounting task, which taps decision-making as well as reward sensitivity (Lamm et al., 2006; MacKillop et al., 2011). The cognitive control and reward sensitivity literature often gives the impression that survey and task measures of these constructs tap the same dimensions and may be interchangeable. However, accumulating evidence suggests they measure different aspects of these constructs (Cyders and Coskunpinar, 2011; Sharma et al., 2014; Zald and Treadway, 2017).

In this paper, we conceptualize cognitive control broadly, consistent with prior literature (Berridge et al., 2009). We use the term executive function (EF) to refer specifically to task-based measures of cognitive control. We adopt the unity and diversity model of EF (Miyake et al., 2000; Friedman and Miyake, 2017), which posits shared variance across EF tasks captured by Common EF alongside separable updating- and shifting-specific variance, with inhibition variance subsumed under Common EF (Friedman and Miyake, 2017). Similar to other conceptualizations of EF, this model addresses the low correlations between individual EF tasks by extracting latent variables that capture what is shared across tasks. In prior studies including those using the ABCD sample, Common EF derived from these tasks has demonstrated stability, biological relevance, and predictive utility for psychopathology (Freis et al., 2022, 2024; Hatoum et al., 2018). Although alternative frameworks distinguish “hot” and “cool” EF, with hot EF reflecting socio-emotional processes and cool EF reflecting cognitive processing (Zelazo and Carlson, 2012; Fernández García et al., 2021), we retain the unity and diversity model due to its validation in ABCD (Freis et al., 2022) and the predominantly “cool” nature of ABCD EF tasks, aside from delay discounting.

Additionally, accruing evidence suggests that reward sensitivity is multidimensional rather than unidimensional (Richards et al., 2016). Distinct facets of reward differentially relate to psychopathology outcomes (Kaiser et al., 2024) and are supported by separate neurobiological mechanisms (Berridge, xbib2018). These facets can be captured through behavioral tasks indexing constructs such as reward learning (Cooper et al., 2014; Kaiser et al., 2024) and reward responsiveness (Van Den Berg et al., 2011), as well as self-report measures assessing dimensions including fun-seeking, drive, and reward responsiveness (Carver and White, 1994). Related constructs in the substance use literature, such as positive urgency and sensation seeking, further underscore the multifaceted nature of reward-related processes (Cservenka et al., 2013; Qianlan et al., 2025).

When studied together, both reward sensitivity and cognitive control appear to have unique survey rating and task performance variance. For example, Harden et al. (2017) used an exploratory structural equation model (ESEM) combining both task-based and self-report survey measures cognitive control and reward sensitivity. They identified four factors —- Cognitive Dyscontrol, Reward-Seeking, Premeditation, and Fearlessness—-with tasks loading on the first two and surveys on the latter two, underscoring the distinct variance each method captures. Studies focusing on cognitive control measures reach similar conclusions: task-based measures (e.g., executive function tasks) and rating-based measures represent phenotypically separable aspects of self-regulation (Gustavson et al., 2023; Freis et al., 2022; Friedman et al., 2020; Duckworth and Kern, 2011).

Importantly, some of these aforementioned studies (Freis et al., 2022; Friedman et al., 2020) showed that task-based and rating-based measures of control independently predict psychopathology (i.e., when controlling for one another). Thus, not only do these different methods capture different variance, but both may be important for understanding psychopathology. Such findings challenge the idea that one method is superior to the other; rather, both task and survey-based measures are relevant to psychopathology.

Given that each method has strengths and weaknesses, researchers have advocated for inclusion of both types of measures in studies of individual differences (Friedman and Banich, 2019). Self-reports may better capture internalized thoughts and feelings that would be difficult to assess otherwise, but they are vulnerable to social desirability bias (Friedman and Gustavson, 2022). While task-based measures provide more objective assessments, they have their own issues, with ecological validity (whether the behavioral tasks are generalizable to real-world scenarios), task reliability, and task impurity (when tasks tap other cognitive processes in addition to the construct of interest) being of particular interest (Cyders and Coskunpinar, 2011; Miyake et al., 2000).

Most prior research only includes a small number of measures and evaluates reward sensitivity and cognitive control in isolation, limiting assessment of their discriminant validity and joint associations with psychopathology. Although considering cognitive control and reward sensitivity separately is frequent in the literature, these domains often overlap at the measurement level. For example, positive urgency reflects impulsive action under positive affect, capturing variance attributable to both reward sensitivity and cognitive control with individual items (e.g., “I tend to act without thinking when I am very, very happy”) tapping both. Similarly, delay discounting tasks index not only sensitivity to immediate rewards but also the regulatory processes required to delay gratification. Evaluating these constructs within a shared measurement framework is therefore essential for partitioning latent variance across domains and strengthening construct validity.

### 1.3 Current study

While previous work regarding task vs. survey divergence has focused on cognitive control, less work has focused on reward sensitivity. We address this gap with an ESEM approach where we not only include task-based measures of both constructs, but also individual items from multiple questionnaires targeting reward sensitivity and cognitive control. This focus on the item level allows us to challenge assumptions of whether these items all capture the same construct (Kozlowski et al., 2025; Harden et al., 2017; Marsh et al., 2014). Moreover, because some items may reflect both cognitive control and reward sensitivity, we include both reward sensitivity and control items in a single ESEM to allow for the possibility that items may load on multiple factors (e.g., some reward sensitivity items might load on factors with control items). Our preregistration hypothesized 4 factors to capture variance relevant to cognitive control and reward sensitivity constructs at both the task-based and survey-based levels.

We then examine the criterion validity of these ESEM factors in relation to psychopathology. In particular, we examine whether the factors we extract via ESEM independently relate to psychopathology, which would support the hypothesis that separable factors capture meaningful differences in construct variance, rather than merely reflecting methodological differences. We address the following questions:

1. Do task-based vs. survey measures of reward sensitivity and cognitive control capture distinct variance at the level of latent variables?
2. If task-based and survey measures capture different variance, do they independently relate to psychopathology? That is, are we able to explain more variance in psychopathology when considering multiple methods of assessing both reward and control during early adolescence?

We further extend prior work by incorporating multiple raters and methods, moving beyond studies relying solely on youth-reports of reward sensitivity and cognitive control (Steinberg, 2010; Harden et al., 2017). This approach is especially important in early adolescence, where self-reported and caregiver-reported measures are thought to be increasingly divergent. The ABCD study is uniquely positioned to examine previously described cross-informant divergence and convergence (Achenbach et al., 1987; De Los Reyes et al., 2015; Hope et al., 1999) because it includes caregiver, teacher, and youth reports.

Past work underscores the value of Multitrait-Multimethod (MTMM) models (Campbell and Fiske, 1959) and multi-informant data to better capture these behaviors across varied contexts (Kozlowski et al., 2025; De Los Reyes and Makol, 2021; Martel et al., 2017). We are particularly interested in whether rater-based measures of reward and control converge across youth and parent reports, or whether factors might separate by rater as well as method. For psychopathology, we have reason to expect moderate convergence across raters based on prior literature (Ready et al., 2002), so we can use multiple raters to create better factors.

The timeframe we examine spans a crucial developmental period (ages 9–12 years) during which cognitive control and reward sensitivity are thought to be related to psychopathology risk (Luna et al., 2010; Urošević et al., 2012; Loso et al., 2021; Ullsperger and Nikolas, 2017). In contrast to past work that has taken a cross-sectional approach (Freis et al., 2022; Harden et al., 2017), we begin to leverage the longitudinal nature of the ABCD study. Doing so guards against inflated relationships due to time-specific effects by relating survey measures of reward and control at the baseline assessment to psychopathological dimensions measured 2 years following. However, though our analyses span multiple timepoints, we are not attempting to establish developmental causal inferences about the direction of relationships between our model specified predictors and outcomes, which would be misplaced given the correlational nature of the data and the possibility of bidirectional associations.

## 2 Method

### 2.1 Transparency and openness

We preregistered the measures and analysis plan. Details can be found at https://doi.org/10.17605/OSF.IO/JKNR5. There is Supplementary material available for this manuscript. We report how we determined our sample size, all data exclusions, all manipulations, and all measures in the study. The studies involving human participants were reviewed and approved by a central institutional review board and comply with the World Medical Association Declaration of Helsinki and APA ethical standards. The University of California San Diego institutional review board has stated that analyses using publicly released ABCD data are not human subjects research and do not require their own protocol approval. Written informed consent to participate in this study was provided by the participants' legal guardian/next of kin. The ABCD data repository grows and changes over time. The ABCD data used in this report came from the ABCD 5.1 data release (doi: HYPERLINK “http://dx.doi.org/10.15154/z563-zd24”).

### 2.2 Participants

Participants included in analyses were 11,866 youth (reported sex 48% female, 52% male; reported age at baseline *M* = 9.92, *SD* = 0.63) from the ABCD study, an ongoing longitudinal study of adolescent development across 21 sites in the United States. Participants are assessed annually, beginning at age 9–10 until age 19–20, including neuroimaging and neuropsychological data, as well as assessments of mental and physical health. Aspects of the protocol are offered at alternating timepoints. The sample was recruited to be demographically (52.2% White; 15.1% Black; 20.4% Hispanic; 3.2% Asian, American Indian/Alaska Native, or Native Hawaiian and other Pacific Islander; 9.2% Multiple races selected) and socioeconomically (Annual family income < $25K 16.1%; $25K–$49K 15.1%; $50K−74K 14.0%; $75K−99K 14.1%; $100K−199K 29.5%; $200K+11.2%) diverse and generally match the American Community Survey national estimates for demographic characteristics (Garavan et al., 2018).

The ABCD study included twins and siblings, so we controlled for non-independence due to relatedness by including family ID as a clustering variable. All analyses were run in Mplus 8.1 (Muthén and Muthén, 1998–2024) using the means and variances-adjusted weighted least squares (WLSMV) estimator, which uses pairwise deletion for missingness. As such, the only participants entirely excluded from analyses are those who were missing on the covariates (age and sex); *n*s for each measure are provided in Supplementary Table S1.

### 2.3 Measures

#### 2.3.1 Reward sensitivity and cognitive control measures

We chose behavioral tasks and survey measures to assess our primary constructs of interest: reward sensitivity and cognitive control. Per our preregistered analysis plan, we used baseline data for all items measured at baseline and year 2 follow-up survey and task measures only for items not administered at baseline (e.g., game of dice task). Delay discounting was only offered at the year 1 follow-up but was included in analyses due to its relevance to reward sensitivity.

We assessed task reward sensitivity using the delay discounting and game of dice tasks. The delay discounting task indexes preference for smaller immediate vs. larger delayed rewards, with greater reward sensitivity reflected in more immediate choices (Koffarnus and Bickel, 2014). Reliability was acceptable (split-half = 0.74; Spearman-Brown prophecy formula). The game of dice task assesses risk-taking in the context of reward by having participants choose from riskier choices with higher rewards vs. safer choices with lower rewards (Pacheco-Colón et al., 2021). The game of dice task showed excellent reliability (split-half = 0.92). We excluded the Monetary Incentive Delay task due poor reliability (split-half = 0.15) of the difference score (mean reaction time of all reward trials–all neutral trials).

The five tasks we included to tap cognitive control were based on prior work evaluating an executive function model (Freis et al., 2022) and included flanker inhibitory control (flanker), stop signal task (SST), emotional n-Back, list sorting working memory, and dimensional change card sort (card sort). Briefly, flanker measures interference control and attention (Luciana et al., 2018), SST measures response inhibition, emotional n-Back measures working memory maintenance and updating (Casey et al., 2018), list sorting working memory measures working memory, and card sort measures cognitive flexibility (Luciana et al., 2018).

We selected individual survey items based on face validity and previous literature irrespective of their original subscale. Items were drawn from the BAS (Carver and White, 1994; fun-seeking and reward-seeking subscales), the UPPS-P (Whiteside and Lynam, 2003; positive and negative urgency, lack of premeditation, and sensation seeking subscales), and the EATQ (Ellis and Rothbart, 2001; activation control, inhibitory control, and high intensity pleasure subscales). These selections were preregistered (https://doi.org/10.17605/OSF.IO/JKNR5). Because we selected individual items from survey measures, we could not assess their internal consistency, however, the overall surveys previously showed good reliability in adolescent samples (Freis et al., 2022; Barch et al., 2018). Descriptive statistics are provided in Supplementary Table S1, with excluded items summarized in Supplementary Table S2.

Individual survey items and their original survey/subscale are provided in Supplementary Table S1. Reliabilities for the reward and cognitive control tasks have been previously reported in ABCD pilot data (Casey et al., 2018; Luciana et al., 2018). Reliability estimates for all tasks in this sample are provided in Supplementary Table S3. More detailed descriptions of the reward and control tasks and descriptive statistics for our sample are provided in Supplementary Table S4.

#### 2.3.2 Psychopathology measures

We used psychopathology measures from the year 2 follow-up because we were interested in whether the ESEM factors measured at baseline would be early indicators of psychopathology measured 2 years later, despite the young age of participants at baseline. Because some individuals were missing data at the year 2 follow-up, we wanted to ensure those individuals did not differ based on psychopathology status at baseline. Baseline psychopathology scores were not statistically significantly different between those missing and not missing data at year 2. More details are provided in the Supplementary methods and full results from this missingness analysis are summarized in Supplementary Table S5.

A caregiver completed the Child Behavior Checklist (CBCL; Achenbach and Verhulst, 2010) at the year 2 follow-up assessments. The CBCL consists of 112 items assessing the presence of behavioral problems (e.g., “Worries”) on a scale of 0 = not true, 1 = somewhat true, and 2 = very true. Items were summed to create subscales for attention, social, thought, somatic, aggressive, rule-breaking, anxious/depressed, and withdrawn/depressed problems. We used the internalizing (sum of somatic, anxious/depressed, and withdrawn/depressed scales) and externalizing (sum of aggressive and rule-breaking scales) composite scores, as well as the attention, social, and thought problems summary scores as separate measures, following prior work in this sample (Freis et al., 2022), which demonstrated that CBCL internalizing and externalizing constructs showed different relationships to task and self-report measures of cognitive control.

The Brief Problem Monitor (BPM; Achenbach and Verhulst, 2010) is an 18-item measure assessing behavioral problems (e.g., “I argue a lot”) and is measured on a scale of 0 = not true, 1 = somewhat true, and 2 = very true. Similar to the CBCL, items were summed to create subscales for attention, internalizing, and externalizing problems. The BPM was administered to youth (BPM-Y) starting at the 6-month follow-up and subsequently at annual timepoints and follow-up phone calls. The BPM teacher form [BPM-T, (Achenbach and Verhulst, 2010)] was also electronically emailed to the youth's primary academic teacher in the weeks following each annual visit.

The Kiddie Schedule of Affective Disorders and Schizophrenia DSM-5 (KSADS-5) was administered to both youth and their caregiver. The KSADS-5 includes modules that provide a diagnostic assessment of disorders such as Bipolar Disorders, Major Depressive Disorder, and Conduct Disorder. Youth and their caregivers were asked to indicate lifetime presence of symptoms as well as current endorsement (i.e., in the last 2 weeks). In the ABCD study the diagnoses and symptoms were scored as 0 = absent or 1 = present and did not include the subthreshold option generally present in KSADS.

The diagnostic classes utilized for this analysis and their corresponding endorsement rates are provided in Supplementary Tables S6–S8. A full list of the KSADS-5 disorders administered and how disorder classes were generated (based on those provided in Duffy et al., 2023) for this analysis is provided Supplementary Table S6. Descriptive statistics for the psychopathology measures are provided in Supplementary Table S8.

### 2.4 Statistical analyses

#### 2.4.1 Data trimming and model estimation

We applied the same data trimming procedure to the cognitive control tasks as Freis et al. (2022). For the card sort, flanker, and list sort NIH Toolbox tasks, we utilized the ABCD recommended age-uncorrected standard scores. Age at baseline and sex were used as covariates in all models. It should be noted that we only used age at baseline because of high collinearity between the age at baseline and age at year 2 (*r* = 0.93). More specific details on data cleaning for the cognitive control tasks are provided in the Supplementary methods.

ESEM analyses used raw data in Mplus 8.1 (Muthén and Muthén, 1998–2024). The TYPE = COMPLEX option was used to account for non-independence of children from the same families and the CATEGORICAL option was used to designate ordinal items. We used the WLSMV estimation method due to the ordinal nature of the survey items. We used the oblique Geomin rotation to allow for correlations between factors.

We treated the CBCL, BPM-Y, and BPM-T scores as continuous in our analyses, square root transformed the sums to reduce skewness, and z-scored them such that they each had a variance of one. We modeled KSADS “diagnoses” as categorial.

Psychopathology scores have previously been shown to be related to sex and age (Achenbach and Ruffle, 2000); we included these covariates as predictors of all latent variables (see Supplementary Table S9 for regression coefficients), as we confirmed that sex and age were related to individual survey items through the ESEM factors (i.e., less parsimonious models in which these covariates directly predicted the indicators did not fit substantially better than more parsimonious models in which the covariates predicted the factors, with RMSEA unchanged, and CFI/SRMR change < 0.005).

To test significance of parameters we used *p-values* for the *z*-tests in the output. For all analyses we used an alpha level of 0.05, after false discovery rate (FDR; Benjamini and Hochberg, 1995) correction for a total of 35 tests: the regressions between the reward sensitivity and cognitive control factors and psychopathology factors as well as the correlations between the reward sensitivity and cognitive control factors.

#### 2.4.2 Model specification: ESEM factors

We conducted an ESEM (Asparouhov and Muthén, 2009) analysis on the reward sensitivity and cognitive control tasks and survey items. The ESEM framework is useful for this study because it allows for an exploratory approach to evaluate how many factors are needed to capture the reward sensitivity and cognitive control data (allowing for indicators to cross-load on multiple factors). However, rather than having to translate the resulting exploratory factors into a confirmatory factor analysis (i.e., force each item to load on one and only one factor) or extract factor scores, ESEM allows for the exploratory latent variables to be directly incorporated into structural equation models. Prior work has shown that this approach can reduce bias in estimation of the relationships of these exploratory factors to other constructs, compared to when these factors are treated in a confirmatory way (i.e., removing “small” cross-loadings; Mai et al., 2018).

We evaluated models with 1 to 9 factors, with model selection based on the pattern matrix, scree plot, and fit statistics, including the root mean square error of approximation (RMSEA), standardized root mean squared residual (SRMR), comparative fit index (CFI). The preregistration for this study (https://osf.io/jknr5) hypothesized 4 factors, and proposed examining models with up to 6 factors, but we evaluated models with more factors after observing an improvement of fit with a higher number of factors extracted. More details on model selection are provided in the Supplementary methods.

#### 2.4.3 Model specification: psychopathology factors

We chose a confirmatory factor analysis (CFA) approach for the psychopathology outcomes because we had a strong hypothesis about how these items should cluster based on prior research (Achenbach and Edelbrock, 1981; Kendler et al., 2003). Details about alternative models can be found in the Supplementary methods.

#### 2.4.4 Model specification: rater factors

Because our both our predictor and outcome latent variables included indicators from multiple raters we accounted for shared rater variance. We did this with a MTMM approach where the “method” factors were confirmatory factors for items from each rater at both stages of the model (e.g., a survey parent rater factor for the reward and control items, and a symptom parent rater factor for the psychopathology items). For each rater factor, we equated the loadings (allowing negative but equated loadings for items with reversed directionality). We equated loadings because we would not expect that any one particular item should load more or less strongly than any other item on each of our rater factors if what is shared between the items is truly due to shared method variance. We additionally allowed for different rater factors for different measure types (individual Likert-rated survey items vs. diagnoses/sum scores of symptoms), with the assumption that the different scaling of the aforementioned measure types is unlikely to result in equal loadings on a single factor. The survey and symptom method factors were allowed to correlate within raters, but not across raters.

**Figure 1:**
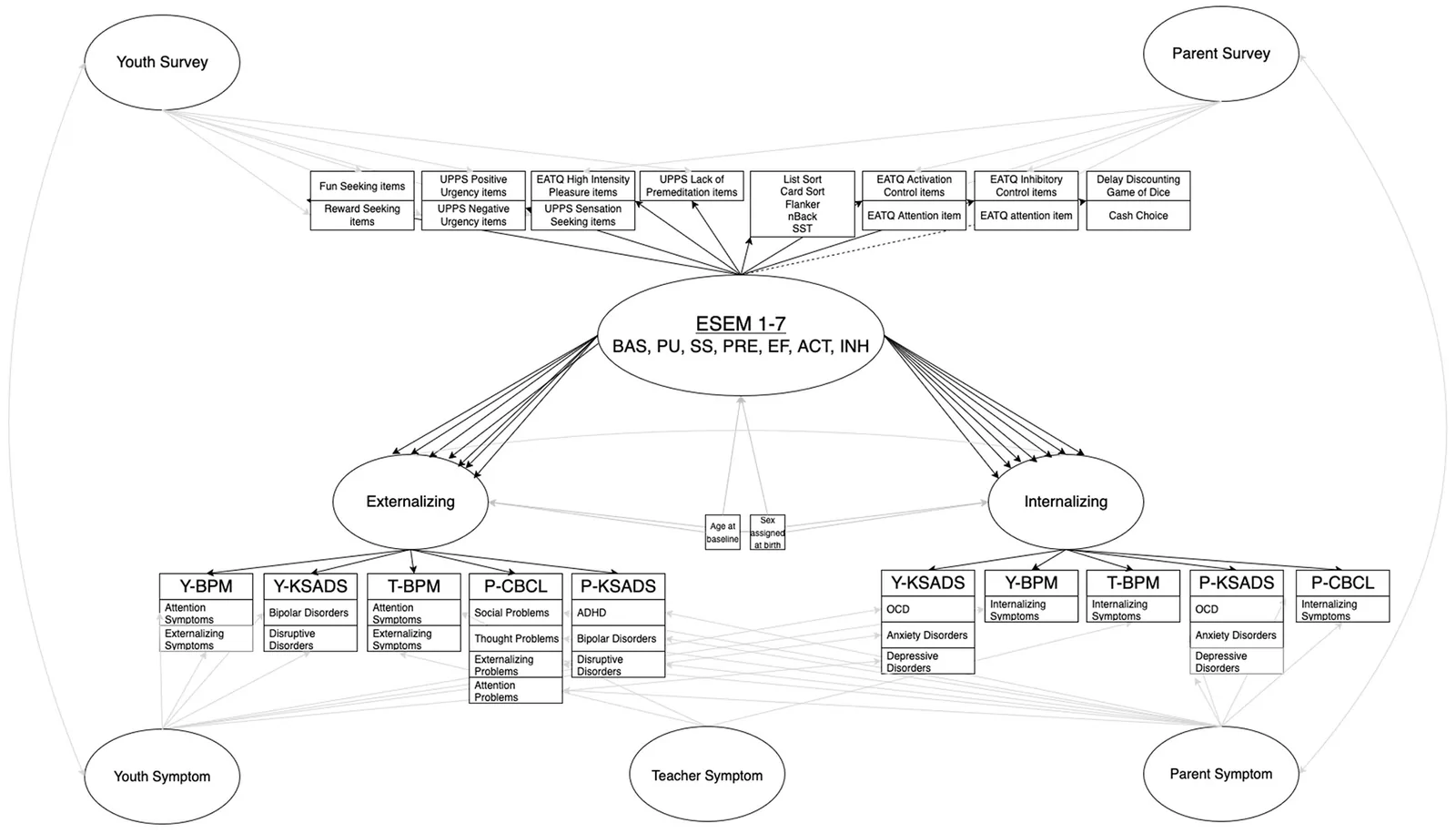
Path diagram representing the Multi-Trait Multi-Method style structural equation model where the method factors are raters. The rectangles represent observed variables/indicators. The ovals represent latent or unobserved variables. The youth and parent survey rater factors and the youth, parent, and teacher symptom rater factors were fixed to be orthogonal to all other factors in the model except for their respective symptom or survey rater factors (e.g., youth survey correlated with youth symptom). This made it such that the path coefficients shown on the unidirectional arrows going from the latent psychopathology and ESEM variables to the indicators are the path coefficients over-and-above their shared variance due to rater. We allowed the Internalizing and Externalizing factors to correlate. Sex and age at baseline were covaried at the factor level. Y-KSADS, Youth-reported Kiddie Schedule of Affective Disorders and Schizophrenia; Y-BPM, Youth-reported Brief Problem Monitor; P-KSADS, parent/caregiver-reported Kiddie Schedule of Affective Disorders and Schizophrenia; P-CBCL, parent/caregiver-reported Childhood Behavior Checklist; T-BPM, Teacher-reported Brief Problem Monitor.

The continuous variables were all scaled to have variances of 1; because the ordinal variables were modeled with a probit link function, they also had variances of 1. Therefore, both the unstandardized and standardized loadings on each rater factor were equated. Figure 1 provides a schematic of the full model.

## 3 Results

### 3.1 Do task-based measures vs. survey measures of cognitive control and reward capture distinct variance?

We used multiple fit criteria to guide model selection, considering standard indices (CFI, SRMR, RMSEA). See Supplementary Table S10 for fit statistics of the 1- through 9-factor solutions.

Because our preregistration hypothesized 4 factors (two reward sensitivity factors—survey and task—and two cognitive control factors—survey and task), we present the loadings for the 4-factor solution in Supplementary Table S11. However, the 4-factor model did not fit well according to the CFI (0.870), Supplementary Table S10. It also did not cluster in the hypothesized manner. Instead, items clustered in an unpredictable manner not supported by other factor solutions. For instance, parent-reported high intensity pleasure items from the EATQ loaded moderately on both F2 and F3 in the 4-factor solution despite F2 showing loadings with the other EATQ subscale items and F3 having loadings from the UPPS lack of premeditation subscale items and task-based EF. Neither the 3 nor 5-factor models showed this pattern of loading, suggesting that this 4-factor solution was not stable.

The 5- and 6-factor models continued to show CFI improvements >0.01. The 7- and 8-factor models were similar to one another with the CFI improving by 0.008 units from 0.939 to 0.947 in the 8-factor model. The RMSEA and SRMR were each smaller by 0.003 units in the 8-factor model compared to the 7-factor model. While we did see these slight improvements in fit statistics going from the 7- to 8-factor model, the interpretation of the factors became less clear in the 8-factor model. The choice between 7- vs. 8-factors was in accordance with our examination of eigenvalues in the scree plot. Given the small change in fit statistics between the 7- and 8-factor models, the more parsimonious and interpretable 7-factor model was chosen. It had acceptable fit (RMSEA = 0.026, SRMR = 0.036, CFI = 0.939).

Due to the large sample, we were able to replicate our ESEM measurement model with two split-sample replication arms. The full ESEM and psychopathology 7-factor model loadings for each replication arm are shown in Supplementary Tables S12 and S13. Briefly, the findings from our split-sample replication reproduced the same factor structure in each replication arm with similar fit. The fit statistics for the 1-through 9-factor solutions for the ESEM model and for the full, final model with psychopathology for each replication arm are shown in Supplementary Table S14.

In the discussion of ESEM results below, our ESEM factor naming scheme mirrors the subscale from which the strongest loading items originated; however, these factors should not be construed as substantively the same as the subscales from which the indicators were derived. The parameter estimates for the final 7-factor model depicted in Figure 1 are shown in Table 1. An overview of these loadings is summarized below. For interpretability this summary focuses on standardized factor loadings ≥0.30, all of which are statistically significant at an FDR-corrected alpha threshold of 0.05. This model suggested that task-based and survey-based measures of cognitive control are distinct. All executive function tasks loaded onto their own factor and had no cross-loadings greater than 0.07. Additionally, cognitive control surveys loaded on more than one factor. Survey-based reward sensitivity measures also loaded on more than one factor, but there was not a task-based reward sensitivity factor; the reward sensitivity tasks did not show substantial loadings on any of the factors, with game of dice showing the strongest loading (λ = 0.17) on the Executive Function task factor.

**Table 1:** Standardized factor loadings and correlations for the seven-factor exploratory structural equation model with psychopathology.

Item	Factor loading							
	Survey/Timepoint (Rater)	BAS (RS)	Executive function tasks (CC)	Lack of premed-itation (CC)	Positive urgency (RS)	EATQ activation control (CC)	Sensation seeking (RS)	EATQ inhibitory control (CC)
I feel excited and full of energy when I get something that I want	BAS BL (self)	**0.63** ^*^	−0.07	0.11	0.06	−0.02	−0.07	0.02
When I am doing well at something, I like to keep doing this	BAS BL (self)	**0.56** ^*^	0.01	−0.16	−0.10	0.02	0.02	0.01
I get thrilled when good things happen to me	BAS BL (self)	**0.68** ^*^	0.01	−0.06	−0.05	0.03	−0.08	−0.02
It would excite me to win a contest	BAS BL (self)	**0.61** ^*^	0.00	−0.03	−0.10	0.00	0.02	0.02
I get really excited when I see an opportunity to get something I like	BAS BL (self)	**0.69** ^*^	−0.07	0.05	−0.02	−0.03	−0.04	0.01
I often do things for no other reason than they might be fun	BAS BL (self)	**0.36** ^*^	−0.02	0.07	0.28	−0.02	0.11	−0.01
I am always willing to try something new, when I think it will be fun	BAS BL (self)	**0.48** ^*^	−0.01	−0.16	0.05	−0.01	0.20	0.00
I crave excitement and new sensations	BAS BL (self)	**0.50** ^*^	0.06	−0.10	0.09	0.01	0.18	−0.04
Cash choice	BL (self)	0.09	−0.02	0.05	0.01	0.03	0.03	−0.05
Opens presents before s/he is supposed to	EATQ Y2 (parent)	−0.02	0.20	0.01	−0.05	−0.03	0.02	**0.45** ^*^
Would be frightened by the thought of skiing fast down a steep slope	EATQ Y2 (parent)	−0.01	−0.07	0.03	−0.03	0.02	**0.51** ^*^	**0.31** ^*^
Usually does something fun for a while before starting her/his homework, even though s/he is not supposed to	EATQ Y2 (parent)	0.00	−0.07	0.02	−0.01	**0.44**	−0.05	0.20
Wouldn't be afraid to try a risky sport like deep sea diving	EATQ Y2 (parent)	−0.01	−0.01	0.04	−0.05	0.11	**0.62** ^*^	0.04
Would like driving a racing car	EATQ Y2 (parent)	0.01	−0.03	0.03	−0.04	0.01	**0.55** ^*^	−0.04
*Wouldn't want to go on the frightening rides at the fair*	EATQ Y2 (parent)	0.04	−0.04	0.04	−0.03	0.04	**0.44** ^*^	0.23
I enjoy taking risks	UPPS BL (self)	0.08	0.18	0.05	0.21	0.01	**0.31** ^*^	−0.04
I like new, thrilling things, even if they are a little scary	UPPS BL (self)	0.17	0.14	−0.07	0.15	−0.03	**0.34** ^*^	−0.02
I would like to learn to fly an airplane	UPPS BL (self)	−0.10	−0.07	−0.29	0.03	−0.06	**0.30** ^*^	−0.03
I would like to ski very fast down a high mountain slope	UPPS BL (self)	−0.01	0.08	−0.14	0.08	−0.05	**0.47** ^*^	−0.01
I often do things on the spur of the moment	UPPS BL (self)	**0.36** ^*^	0.02	0.06	0.25	0.00	0.15	−0.02
*Has a hard time finishing things on time*	EATQ Y2 (parent)	0.03	0.08	−0.01	0.03	**0.57** ^*^	−0.01	**0.35** ^*^
*Has a hard time waiting his/her turn to speak when excited)*	EATQ Y2 (parent)	−0.07	−0.02	−0.12	0.05	0.01	−0.02	**0.55** ^*^
When asked to do something does it right away, even if s/he doesn't want to	EATQ Y2 (parent)	−0.03	−0.05	−0.06	0.01	**0.46** ^*^	0.03	0.14
*Is more likely to do something s/he shouldn't do the more s/he tries to stop her/himself*	EATQ Y2 (parent)	0.01	0.15	−0.01	−0.04	0.07	0.04	**0.50** ^*^
Usually finishes her/his homework before it's due	EATQ Y2 (parent)	−0.01	0.06	0.00	−0.03	**0.80** ^*^	0.00	−0.15
Usually gets started right away on difficult assignments	EATQ Y2 (parent)	−0.02	−0.03	−0.01	0.00	**0.94** ^*^	0.02	−0.19
*Usually puts off working on a project until it is due*	EATQ Y2 (parent)	0.02	−0.08	0.02	0.02	**0.75** ^*^	−0.03	0.07
Is able to stop him/herself from laughing at inappropriate times	EATQ Y2 (parent)	−0.06	0.15	−0.03	−0.06	0.07	0.09	0.07
*Is often in the middle of doing one thing and then goes off to do something else without finishing it*	EATQ Y2 (parent)	0.03	0.02	0.02	0.01	**0.40** ^*^	−0.04	**0.42** ^*^
Is usually able to stick with his/her plans and goals	EATQ Y2 (parent)	−0.05	0.12	−0.04	−0.02	**0.50** ^*^	0.07	0.08
*I like to stop and think about things before I do it*	UPPS BL (self)	0.00	−0.04	**0.71** ^*^	0.01	−0.01	−0.01	−0.02
When I feel bad, I often do things I later regret in order to make myself feel better now	UPPS BL (self)	0.03	−0.07	−0.12	**0.36** ^*^	−0.01	−0.04	0.01
*I try to take a careful approach to things*	UPPS BL (self)	−0.12	−0.03	**0.53** ^*^	−0.03	−0.04	0.12	0.03
When I am upset I often act without thinking	UPPS BL (self)	0.08	0.11	0.20	**0.38** ^*^	0.01	−0.06	−0.09
I tend to get things done on time	UPPS BL (self)	−0.09	−0.07	**0.36** ^*^	0.03	−0.27	−0.03	0.01
*I am very careful*	UPPS BL (self)	−0.03	0.06	**0.57** ^*^	0.03	−0.04	0.13	−0.01
*I tend to stop and think before doing things*	UPPS BL (self)	0.01	0.05	**0.80** ^*^	0.04	0.02	0.01	−0.01
When I am in a great mood, I tend to do things that can cause me problems	UPPS BL (self)	−0.15	0.03	−0.01	**0.70** ^*^	0.01	0.02	−0.01
I tend to act without thinking when I am very, very happy	UPPS BL (self)	0.02	−0.11	−0.04	**0.69** ^*^	0.00	−0.01	0.02
When I get really happy about something, I tend to do things that can lead to trouble	UPPS BL (self)	−0.08	0.01	0.05	**0.79** ^*^	0.00	0.02	0.01
I tend to lose control when I am in a great mood	UPPS BL (self)	0.02	−0.11	−0.01	**0.69** ^*^	0.00	−0.01	0.02
Game of dice	Task Y2	−0.03	0.17	−0.01	−0.05	0.05	−0.02	−0.01
Delay discounting- ln(k)	Task Y1	0.02	−0.12	−0.01	0.08	−0.01	−0.06	0.02
Flanker	Task BL	0.01	**0.58** ^*^	0.02	0.01	−0.02	−0.01	0.00
List sort	Task BL	−0.02	**0.54** ^*^	0.05	−0.04	0.04	0.00	0.02
Card sort	Task BL	−0.01	**0.64** ^*^	−0.02	0.00	0.03	−0.03	0.00
Nback	Task BL	−0.02	**0.53** ^*^	0.01	0.00	0.00	−0.01	0.05
SST	Task BL	−0.01	−0.25	0.03	0.00	0.01	0.07	−0.05
**Factor correlations**								
BAS		1						
EF		−0.04^*^	1					
PU		−0.19^*^	0.05^*^	1				
PRE		0.18^*^	−0.13^*^	0.20^*^	1			
ACT		−0.05^*^	0.19^*^	−0.16^*^	−0.18^*^	1		
SS		0.06^*^	0.07^*^	0.07^*^	0.09^*^	0.06^*^	1	
INH		−0.11^*^	0.07^*^	−0.08^*^	−0.16^*^	0.41^*^	−0.02	1

Not shown are the parent and youth rater factors, which each had equated loadings from each item from that rater. BL, Baseline; BAS, Behavioral Activation Scale; UPPS, Urgency, Premeditation, Perseverance, Sensation-Seeking; EATQ, Early Adolescent Temperament Questionnaire; EF, Executive Function; Y2, Year 2 Follow-up; RS denotes a reward sensitivity factor; CC denotes a cognitive control factor; Factor loadings with absolute values >0.30 are highlighted in bold. ^*^p < 0.05 after FDR correction; Italics denotes a reverse-coded item.

The first factor was composed of BAS items from both the reward responsiveness and fun seeking subscales, both of which were self-reported. We categorized this factor as a reward sensitivity factor. The highest loading item from the reward responsiveness subscale was, “I get really excited when I see an opportunity to get something I like” (λ = 0.69). The highest loading item from the fun seeking subscale was, “I crave excitement and new sensations” (λ = 0.50). In addition, loadings for items from other scales were small with none exceeding λ = 0.17. Given this pattern of loadings, we named this factor BAS.

The second factor, which we named Executive Function, was differentiated from the other 6 factors because it was comprised of the cognitive control behavioral tasks rather than surveys. We categorized this factor as a cognitive control factor. All cognitive control tasks except for SST loaded at 0.50 or greater on this factor. Other loadings for this factor were small with none exceeding 0.17.

We named the third factor Lack of Premeditation, because it had loadings of 0.40 or greater for all self-reported UPPS-P lack of premeditation items. We categorized this factor as a cognitive control factor. Loadings from other scales were small overall with the highest being 0.36 from the item, “I tend to get things done on time” from the UPPS-P lack of perseverance subscale. The lack of perseverance and premeditation subscales of UPPS-P have been shown to be moderately correlated (Cyders et al., 2014).

The fourth factor, which we named Positive Urgency, consisted of items from the self-reported UPPS-P survey, particularly from the positive urgency subscale, which assesses the tendency to act rashly in the face of strong positive emotions (Lynam, 2013). We categorized this factor as a reward sensitivity factor. The highest loading item on this factor was, “When I get really happy about something, I tend to do things that can lead to trouble” (λ = 0.79). The two negative urgency items also loaded on this factor with the highest negative urgency item being “When I feel bad, I often do things I later regret in order to make myself feel better now,” (λ = 0.36). It has previously been shown that the positive urgency and negative urgency scales moderately correlate (Cyders et al., 2014).

We named the fifth factor Activation Control because it showed loadings of 0.40 or higher for all items from the informant-reported EATQ activation control subscale, with the highest loading for “Usually gets started right away on difficult assignments” (λ = 0.94). We categorized this factor as a cognitive control factor. The activation control subscale has been used to describe behavior related to performing difficult or unpleasant actions, sometimes called procrastination (Ellis and Rothbart, 2001). Overall, loadings from other scales were small with most < |λ = 0.27|, apart from two items that had a loadings of 0.40 and 0.50 that were thematically related to task completion: the first item, “Is often in the middle of doing one thing and then goes off to do something else without finishing it” came from the attention subscale of the EATQ; the second item, “Is usually able to stick with his/her plans or goals,” came from the EATQ inhibitory control subscale. One of these activation control items also cross-loaded onto the seventh factor (λ = 0.42).

The sixth factor, which we named Sensation Seeking, consisted of a mix of surveys and raters (self vs. informant) with the common thread being risk-taking behavior. We categorized this factor as a reward sensitivity factor. Items loading at 0.30 or greater came from the parent-reported EATQ high intensity pleasure subscale and the youth-reported UPPS-P sensation seeking subscale. Participants scoring highly on this factor reported willingness to sea dive and ski fast down high mountain slopes.

The seventh factor, which we named Inhibitory Control, was also comprised of informant-reported EATQ items, but primarily from the inhibitory control subscales. We categorized this factor as a cognitive control factor. The inhibitory control subscale measures one's ability to plan and suppress inappropriate responses. All inhibitory control items loaded at 0.40 or greater except for two items: “Is usually able to stick with his/her plans and goals” loaded more strongly onto the Activation Control factor, and “Is able to stop him/herself from laughing at inappropriate times” loaded at 0.07 on this factor. This item did not load highly on any factor. This seventh factor included a loading of 0.42 for the attention subscale item, “Is often in the middle of doing one thing and then goes off to do something else without finishing it” that also loaded onto the Activation Control factor. Given that procrastination and sensation seeking have both been related to impulsive action and all have been related to attention problems (Faraone et al., 2021; Patros et al., 2016; Erskine et al., 2013; Gustavson et al., 2014), it is not surprising that this item had significant loadings on both factors. This factor also included a loading of 0.31 for the EATQ high intensity pleasure item, “Would be frightened by the thought of skiing fast down a steep slope.”

The factor correlations displayed predictable patterns, with the strongest correlation between informant-reported Activation Control and Inhibitory Control (*r* = 0.41). The next strongest correlation was between self-reported Positive Urgency and Lack of Premeditation factors (*r* = 0.20). Informant-reported Activation Control showed a small-moderate relationship with task-based Executive Function (*r* = 0.19), consistent with a prior study relating a common factor for EATQ to a common factor for executive function (Snyder et al., 2021). This relationship was the strongest between any of the survey-based cognitive control factors and the Executive Function factor, with the rest ranging from *r* =|0.05–0.13| (all *p* < 10^−4^).

### 3.2 Relations to psychopathology

Zero-order correlations among psychopathology measures are shown in Supplementary Figure S1, with rater and psychopathology factor loadings reported in Supplementary Table S15. Briefly, the psychopathology factors include items from both self- and informant-reported psychopathology from the BPM, CBCL, and KSADS. The final two-factor CFA solution for Internalizing and Externalizing demonstrated acceptable model fit (Supplementary Table S16). Alternative models are presented in the Supplementary methods.

To evaluate whether the ESEM latent variables tapping similar constructs would independently relate to psychopathology, we added psychopathology factors as outcomes predicted by the seven ESEM factors. We examined relationships between the ESEM factors and psychopathology factors over and above variance due to rater and method by creating CFA rater factors in both portions of the model (Figure 1). For rater method factors, item loadings were equated such that higher scores reflected greater impulsivity and reward sensitivity, and rater factors were allowed to correlate across the ESEM and psychopathology components. Loadings on the survey rater factors were generally small but significant (λ < |0.30|), suggesting some shared variance due to rater effects (Widaman, 2018). Loadings on the symptom rater factors were larger (λ = 0.45–0.57). Correlations between the corresponding survey and symptom rater factors were moderate (*r* = 0.44 for youth, *r* = 0.35 for parent).

**Figure 2:**
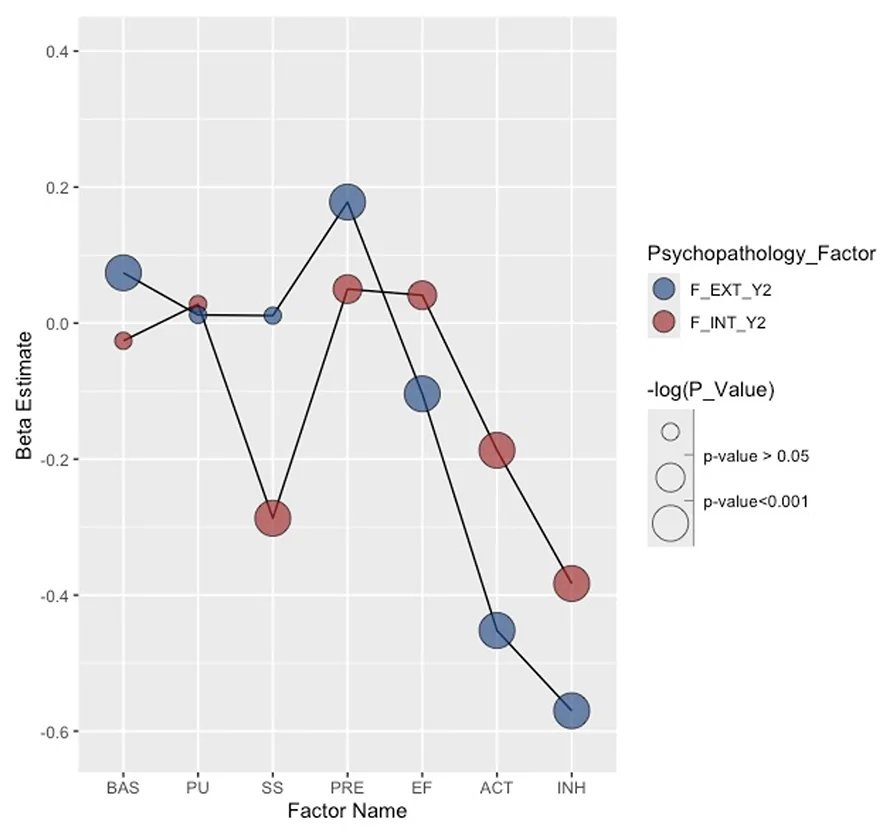
Regression coefficient estimates of seven-factor exploratory structural equation model factors predicting Externalizing and Internalizing disorders factors. BAS, BAS factor; PU, Positive Urgency factor; PRE, Lack of Premeditation factor; SS, Sensation Seeking factor; EF, Executive Function factor; ACT, Activation Control factor; INH, Inhibitory Control factor. F_EXT_Y2, Externalizing disorders factor with externalizing psychopathology indicators all measured at year 2 follow-up timepoint. F_INT_Y2, Internalizing disorders factor with internalizing psychopathology indicators all measured at year 2 follow-up timepoint. All *p-values* were FDR corrected.

The patterns of standardized regression coefficients of these two psychopathology factors on the 7 ESEM factors are shown in Figure 2. These regression coefficients estimate the unique prediction of each ESEM factor, controlling for the other ESEM factors. For comparison, in Table 2 we also present the correlations of the ESEM factors with psychopathology factors, which estimate the relation between each ESEM factor and psychopathology without controlling for variance shared with other ESEM factors. Relations between the seven ESEM factors and individual psychopathology measures/diagnoses are shown in Supplementary Figure S2. Raw correlations among the psychopathology measures are shown in Supplementary Table S17 and Supplementary Figure S1.

**Table 2:** Comparison between psychopathology relationships with reward sensitivity and cognitive control factors in a multiple regression model and a correlational models.

ESEM Factor Predictor	Multiple regression model				Correlational model			
	β to internalizing	SE	β to externalizing	SE	*r* with internalizing	SE	*r* with externalizing	SE
BAS	−0.03	0.017	0.07^*^	0.013	0.001	0.016	0.13^*^	0.014
PU	0.03	0.016	0.01	0.012	0.10^*^	0.016	0.25^*^	0.014
PRE	0.05^*^	0.017	0.18^*^	0.014	0.11^*^	0.016	0.29^*^	0.014
SS	−0.29^*^	0.017	0.01	0.013	−0.28^*^	0.018	−0.03^*^	0.017
EF	0.04^*^	0.017	−0.10^*^	0.014	−0.04^*^	0.017	−0.22^*^	0.015
ACT	−0.19^*^	0.020	−0.45^*^	0.021	−0.36^*^	0.019	−0.74^*^	0.021
INH	−0.38^*^	0.021	−0.57^*^	0.025	−0.46^*^	0.017	−0.79^*^	0.014

ESEM, exploratory structural equation model; BAS, Behavioral Activation Scale; PU, Positive Urgency; PRE, Lack of Premeditation; SS, Sensation Seeking; EF, Executive Function; ACT, Activation Control; INH, Inhibitory Control; SE, standard error.

^*^p < 0.05 after FDR correction.

#### 3.2.1 Do multiple measures of cognitive control and reward sensitivity independently predict psychopathology?

In the results below, it is important to remember that while we include INT and EXT as outcomes predicted by ESEM factors, these models, like other multiple regression models, capture covariation and should not be interpreted to imply causation. Cognitive control factors, measured via tasks and informant- or self-reported surveys, were independently associated with both Externalizing and Internalizing psychopathology. Reward sensitivity constructs also showed independent associations, though effects were weaker overall. Standardized regression coefficients for ESEM factors predicting Internalizing and Externalizing are presented in Figure 2. With the large sample size, we had power to detect small effects; Monte Carlo simulations in Mplus indicated 80% power to detect correlations as small as *r* = 0.05 (see Supplementary methods). We highlight standardized paths larger than |0.10|, as effects in this range have been shown in prior work using task and rater measures of cognitive control (Friedman et al., 2020; Gustavson et al., 2023). Associations between ESEM factors and individual psychopathology measures are shown in Supplementary Figure S2.

The Externalizing factor was significantly related to cognitive control factors measured by task and survey. The Externalizing factor showed a statistically significant negative relationship with the Executive Function factor (β = −0.10; *p* < 10^−4^), consistent with prior work (Freis et al., 2022; Friedman et al., 2020). As can be seen in Figure 2, survey measures of cognitive control also showed significant relationships with externalizing psychopathology across rater and measure, controlling for their shared variance with task-based measures of cognitive control. Two of the largest effects between the ESEM factors for control and the Externalizing factor were between Externalizing and Activation Control (β = −0.45; *p* < 10^−4^) and Inhibitory Control (β = −0.57; *p* < 10^−4^), though Externalizing's relationship with the Lack of Premeditation factor was also significant (β = 0.18; *p* < 10^−4^).

Relationships between the Externalizing factor and ESEM factors measuring reward sensitivity (BAS, Positive Urgency, and Sensation Seeking) were not as strong. The Externalizing factor was significantly positively related to BAS (β = 0.07; *p* < 10^−4^). The Externalizing factor was not significantly related to Positive Urgency or Sensation Seeking (β = 0.01; *p* = 0.376; β = 0.01; *p* = 0.409, respectively). This result contrasts with previous work that has shown a strong and positive relationship between externalizing psychopathology and sensation seeking behavior. However, it is consistent with a non-significant relationship between externalizing behaviors and positive urgency once controlling for other impulsivity facets (Kendler and Myers, 2013; Carlson et al., 2013; Mann et al., 2017).

The relationship between the Internalizing factor and cognitive control factors were mixed. The relationship between Executive Function and the Internalizing factor was near zero and not statistically significant (β = 0.04; *p* = 0.026). In contrast to the weak association with the task-based Executive Function factor, the Activation Control and Inhibitory Control factors showed small to moderate, statistically significant, negative relationships with internalizing psychopathology (β = −0.19; *p* < 10^−4^; β = −0.38; *p* < 10^−4^, respectively). However, the Lack of Premeditation cognitive control factor displayed only a small relationship to the Internalizing factor (β = 0.05; *p* = 0.006).

Two of the reward sensitivity factors displayed near-zero relationships with the Internalizing factor, β = −0.03; *p* = 0.142 for BAS and β = 0.03; *p* = 0.112 for Positive Urgency. However, the Internalizing factor did display a small-moderate negative relationship with Sensation Seeking (β = −0.28; *p* < 10^−4^). Supplemental Table S18 includes sensitivity analyses to probe the relationship between anhedonic symptoms and reward sensitivity specifically. Findings did not differ significantly from the broader CBCL internalizing scale.

In addition to our multiple regression results presented above, we also examined a model where the shared variance between these factors is not controlled for by instead allowing for the reward sensitivity and cognitive control factors to correlate with the psychopathology factors. This model is shown in Table 2. As can be seen by the strength of the relationships in the correlational vs. multiple regression model there is attenuation in almost all cases in the multiple regression. This finding is particularly relevant when considering the ESEM framework where items are allowed to cross-load. The fact that the correlational model shows attenuation in effects compared to the multiple regressions suggests that there is shared variance across these factors, suggesting some convergent validity.

### 3.3 Alternative CFA model

Because some factors appeared to cluster by subscale, we wondered to what extent our final model is similar to a CFA with factors corresponding to a priori subscales. In Supplementary Tables S19 and S20, we present results of a CFA in which items loaded only on their original survey subscales (e.g., BAS reward-seeking vs. fun-seeking) or task-specific factors for reward and control tasks. Cash choice was allowed to load with reward sensitivity task factor and the EATQ attention item was loaded onto the Activation Control factor based on where they loaded highest in the ESEM. We retained orthogonal rater factors at the level of both cognitive/reward and psychopathology. Compared to our final model, this approach included 11 subscale factors instead of 7 ESEM factors.

The model correlating these subscale factors with psychopathology showed some convergence with the 7-factor ESEM results, see Supplementary Table S20. In particular, these subscale factors showed very similar patterns and magnitudes of correlations with Internalizing and Externalizing psychopathology as the ESEM factors that corresponded with subscales did (Table 2). As in the ESEM, parent-reported EATQ scales for inhibitory control and activation control showed the largest (negative) associations with Externalizing psychopathology. In addition, the task-based Executive Function factor was related to both Internalizing and Externalizing factors, but showed a stronger correlation to the latter. However, in this model, the relationship of the CFA task-based reward factor to Externalizing psychopathology was high, which should be considered with caution given the very low standardized loadings of the tasks on this factor (0.21 and −0.14). Of note, this CFA task-based reward factor was also strongly correlated with the task-based Executive Function factor (*r* = 0.87, see Supplementary Table S19), consistent with the ESEM finding that the reward tasks loaded most strongly with the EF tasks (although these reward task loadings were still small, as shown in Table 1).

This model showed a poorer fit compared to the ESEM [**χ^2^**(2,308) **=** 38,908.931; *p* < 0.001; CFI = 0.871; RMSEA = 0.037; SRMR = 0.050], unsurprisingly given that the ESEM allows cross-loadings whereas the CFA does not. In addition, the model regressing psychopathology factors on these 11 subscale factors resulted in a solution with a non-positive definite latent variable covariance matrix and implausibly high and counterintuitively positive regression coefficients for multiple ESEM factors to psychopathology factors and large standard errors around these regression estimates (Supplementary Table S20). These problems likely at least partially reflect the fact that the CFA showed high correlations among some subscales that were collapsed into single factors in the ESEM, alleviating multicollinearity problems when regressing psychopathology factors onto these reward and control factors.

One way to address this multicollinearity would be to include higher-order factors to capture shared variances across subscales. We tried a model in which we used higher-order factors for each instrument (i.e., a higher-order BAS factor on which the two BAS subscales loaded; a higher-order UPPS-P factor, and a higher-order EATQ factor), along with the Executive Function and Reward Task factors and correlated psychopathology factors. This model fit worse than the model without the hierarchical factors [**χ^2^**(2,368) **=** 45,853.992; *p* < 0.001; CFI = 0.847; RMSEA = 0.039; SRMR = 0.057]. The version of this hierarchical model in which the psychopathology factors correlated with these five factors, as well as a version in which the psychopathology factors were regressed on these five factors still resulted in a non-positive definite covariance matrix, and the unreasonably high correlation and regression estimate between the reward task factor and Internalizing psychopathology (Supplementary Table S21) also remained (*r* = 0.602 and ß = 0.831). This CFA clustering is not identical to the clustering in the ESEM, because it focused on factors for the instruments, rather than constructs. For example, in the ESEM, items from the parent-reported EATQ high intensity pleasure subscale and the youth-reported UPPS-P sensation seeking subscale loaded together, whereas they were separated in the CFA models. Moreover, the high correlation between the Executive Function and Reward Task factors is not captured by a hierarchical instrument factor; we would not have had them loading on a higher-order factor a priori given the goals of the study, so doing so would be a *post-hoc* decision based on the results of the ESEM.

Taken together, the results of this exploration of alternative CFA models provide some assurance that the ESEM factors we examined in the primary model are reasonably aligned with the constructs tapped by previously validated subscales from these instruments. Our ESEM factors correlated similarly to the subscale CFA factors that included similar items. At the same time, this analysis also reveals that the ESEM approach suggested a slightly different factor structure across items spanning different subscales than one simply driven by the original scales, combining items across some subscales but not others. Overall, although the ESEM ended up with somewhat similar factors to subscales, the value of the ESEM approach is that it enables a data-driven exploration of the extent to which the items from these different subscales and different raters would load together.

## 4 Discussion

We found that cognitive control and reward sensitivity are each multidimensional. After controlling for rater effects, we found multiple cognitive control factors (Lack of Premeditation, Activation Control, Inhibitory Control, and Executive Function) and multiple reward sensitivity factors (BAS, Sensation Seeking, Positive Urgency, but no reward sensitivity task factor). These factors were independently associated with Internalizing and Externalizing psychopathology.

### 4.1 Task-based vs. survey-based measures of cognitive control and reward sensitivity capture distinct variance at latent variable level

After controlling for rater variance, we found that task- and survey-based measures formed distinct latent factors. Self/informant-reported cognitive control only weakly correlated with task-based control, providing evidence for discriminant validity and consistent with prior work (Freis et al., 2022; Friedman et al., 2020; Harden et al., 2017). This result highlights the importance of considering multiple methods. Task-based and survey-based measures of a construct are often assumed to be interchangeable, but our findings add to a growing body of literature suggesting they likely tap different facets of these constructs.

We found evidence for the multidimensionality of reward sensitivity. The survey-based reward factors (BAS and Sensation Seeking) appeared to capture distinct variance and were minimally correlated. This pattern suggests they capture unique variance, which is in line with literature that has suggested reward sensitivity as a component that underlies sensation seeking but that is phenotypically separable from sensation seeking (Harden et al., 2018; Castellanos-Ryan et al., 2011). Moreover, we did not observe a distinct reward task factor, raising questions of construct validity. Ideally, reward tasks should correlate with each other and with survey-based reward measures more strongly than with unrelated constructs; instead, they loaded most strongly on the Executive Function factor (Table 1). This pattern could indicate poor construct validity, but prior work shows task- and survey-based measures of reward sensitivity and cognitive control often diverge (Harden et al., 2017; Friedman and Gustavson, 2022; Zald and Treadway, 2017). They may capture unique aspects of these constructs, as evidenced by their concurrent associations with psychopathology (Friedman et al., 2020).

Task selection may also contribute: unlike prior studies employing tasks such as the Iowa Gambling Task (Harden et al., 2017), the ABCD study included delay discounting, which relates to both reward sensitivity and cognitive control (Weigard et al., 2014; Van den Bos et al., 2015). Delay discounting did not cross-load on reward factors, as hypothesized, but instead loaded at λ = 0.17 on the Executive Function task factor. This clustering with the Executive Function task factor further supports work suggesting delay discounting may be more accurately characterized as a cognitive control rather than reward sensitivity task (Fernández García et al., 2021; Lamm et al., 2006) and underscores the importance of considering exploratory rather than solely confirmatory frameworks in examining these correlated constructs. Finally, it is important to note that while we examined reward sensitivity prior to the hypothesized peak in mid-late adolescence, we still see meaningful individual differences in reward (e.g., Sensation Seeking's statistically significant negative relationship to INT), though we would expect these relationships between reward sensitivity constructs and psychopathology to strengthen through adolescence.

Although our motivation for including reward sensitivity and cognitive control in the same ESEM was to examine whether items that might index both reward sensitivity and cognitive control (e.g., delay discounting and UPPS positive urgency) would show cross-loadings indicative of their construct overlap, neither the delay discounting task nor the Positive Urgency factor cross-loaded meaningfully. The Positive Urgency factor consistently emerged as a distinct subconstruct, even in 4- and 5-factor solutions, with no significant cross-loadings on other factors.

While aspects of our final model resembled existing subscales, there were notable exceptions. Most strikingly, parent-reported high-intensity pleasure items clustered with youth sensation-seeking items, forming a cross-instrument, cross-rater factor that related to Internalizing psychopathology. More predictably, positive and negative urgency items clustered together, as did items from the two BAS subscales. Finally, we observed some construct-level cross-loadings: for instance, BAS fun-seeking items showed small cross-loadings (λ < 0.30) on the Positive Urgency factor, suggesting shared reward sensitivity variance. We considered an alternative CFA “subscale” model, in which items loaded onto confirmatory factors based on their original subscales and tasks loaded onto separate task factors, along with the orthogonal rater method factors of our final model. Despite having more factors (11 total), this model showed worse fit, and it yielded implausible relationships to psychopathology (with large standard error estimates), indicating poor model performance.

### 4.2 Multiple measures of reward sensitivity and cognitive control relate to psychopathology

Many of these latent variables were independently related to different psychopathology dimensions, suggesting that they may tap meaningfully different variance in reward and control. Once controlling for shared variance across reward and control subconstructs, almost all factors showed significant relationships to Internalizing or Externalizing. Some of these patterns differed across psychopathology construct.

The Executive Function factor was negatively associated with the Externalizing factor, consistent with prior work (Friedman et al., 2020). In contrast to prior work suggesting associations with internalizing, such as depression (Snyder, 2013), in this sample the Executive Function factor showed no overall association with the Internalizing factor. Disorder-specific analyses revealed a more nuanced pattern: anxiety symptoms (youth- and parent-rated) were positively related to executive function, whereas depression symptoms were negatively or not significantly related (Supplementary Figure S2). These findings are consistent with evidence that anxiety disorders are not reliably linked to executive deficits (Berggren and Derakshan, 2013; Smitherman et al., 2007) and that accounting for externalizing comorbidity can reveal positive associations between executive function and internalizing symptoms (Freis et al., 2022; Clark et al., 2021; Caspi et al., 2014). However, effect sizes were small (*rs* < 0.12), limiting replicability in smaller samples, and associations may strengthen later in adolescence, as has been suggested by age-moderation effects (Snyder et al., 2019).

Other cognitive control relationships to psychopathology were in line with previous work (Buckholtz and Meyer-Lindenberg, 2012; Eisenberg et al., 2009; McTeague et al., 2016): cognitive control factors as measured by survey items exhibited transdiagnostic properties. For instance, Inhibitory Control and Activation Control showed evidence for transdiagnostic associations, though their relationships to the Externalizing factor were nominally larger than their relationships with the Internalizing factor. Beyond the transdiagnostic relationships with cognitive control measures, we found other cognitive control factor results consistent with previous literature. Lack of Premeditation (youth-report) and Inhibitory Control (parent-report) were both significantly and independently related to the Externalizing psychopathology factor, consistent with prior literature on cognitive control (Nigg, 2006).

Our findings surrounding reward sensitivity were not as clearly consistent with prior literature as those for cognitive control. First, although the Positive Urgency factor was positively associated with both Internalizing and Externalizing factors, the effect sizes were smaller than those reported in older samples. Past literature has suggested that UPPS urgency items, both positive and negative, show the strongest associations with internalizing and externalizing disorders (Berg et al., 2015; Friedman et al., 2020; Gustavson et al., 2020, 2023). One possibility for this discrepancy is that it is developmental: positive urgency may not be fully expressed in early adolescence. Supporting this view, correlates of reward sensitivity such as risk-taking typically peak in mid-late adolescence (Cyders et al., 2007). These associations may strengthen with age.

Second, we found only weak evidence that the BAS factor was related to Externalizing symptoms, limiting conclusions about whether higher reward sensitivity corresponds to greater externalizing pathology. The Sensation Seeking factor also showed mixed patterns: it was unrelated to the Externalizing factor, diverging from prior work linking sensation seeking to ADHD (Nigg, 2006) and substance use disorders (Tervo-Clemmens et al., 2020), but negatively associated with Internalizing, consistent with theories that internalizing disorders reflect lower reward sensitivity. Again, developmental timing may partly explain these patterns, as reward sensitivity—including sensation seeking—tends to increase across adolescence and peak in mid-late adolescence (Urošević et al., 2012; Steinberg et al., 2018). Thus, as with Positive Urgency, associations of BAS and Sensation Seeking with psychopathology may strengthen with age.

### 4.3 Psychopathology shows relations to cognitive control and reward sensitivity beyond effects due to rater

Finally, we examined convergent and divergent patterns between our seven ESEM factors and youth, parent, and teacher ratings of psychopathology, given evidence that multiple informants capture distinct symptom manifestations across contexts (De Los Reyes et al., 2015). Consistent with prior work, externalizing disorders showed stronger cross-rater convergence than internalizing (Achenbach et al., 1987). Although some ESEM factors were confounded with rater (e.g., BAS, EATQ), extracting orthogonal rater factors allowed us to account for shared rater variance. Utilizing the same multi-trait multi-rater approach with our psychopathology measures also enabled the extraction of cross-rater psychopathology factors. Results suggest that traits such as Inhibitory Control may have transdiagnostic associations beyond variance due to rater, consistent with prior findings (Arnatkeviciute et al., 2023; Snyder et al., 2015; Goldstein and Volkow, 2011).

Our factor analytic model of psychopathology did not find strong evidence suggesting the need for a 3rd “thought disorders” factor in this sample at this age (see Supplementary methods). While past literature in separate age groups and samples (Caspi et al., 2014) suggests that there is variance unique to this class of disorders, previous polygenic score (PGS) work in the ABCD sample has found that thought disorder symptomology, such as that measured by the Prodromal Psychosis Scale, is more genetically related to internalizing disorders than it is to thought disorders (Paul et al., 2023). It may be the case that in this age group the primary symptoms are manifesting as internalizing symptoms.

### 4.4 Strengths, limitations, and future directions

This study had multiple strengths including the use of the large and demographically diverse ABCD study, which also provided the benefit of being able to assess relationships among constructs across time, and across raters, and methods. Furthermore, our utilization of ESEM provided notable strengths such as being able to take an exploratory approach in determining the number of factors necessary to represent the data. ESEM also offers the benefit of allowing cross-loadings on multiple factors, which reduces bias in the correlations between the factors compared to a model that assumes no cross-loadings (Asparouhov and Muthén, 2009; Marsh et al., 2014). This characteristic is particularly helpful in this model where items may tap both reward sensitivity and cognitive control constructs. Though some argue that exploratory analyses such as ESEM are less reproducible, we show that our factor structure remained stable in split sample replications which reproduced same factor structure with almost identical fit (Supplementary Tables S12–S14). Lastly, endorsement of psychopathology in this sample is similar to national estimates (Cosgrove et al., 2011; Danielson et al., 2021), meaning that in contrast to high-risk samples, the psychopathology relationships to the ESEM factors may represent effects seen in the early adolescent general population.

Our study is not without limitations. First, we found generally small effect sizes between ESEM factors and psychopathology, with only Inhibitory Control exceeding β = |0.40|. These effect sizes align with prior work in the ABCD sample showing median effects around 0.05 (Owens et al., 2021) and with meta-analytic findings that typical psychological effects average *r*~0.19 (Gignac and Szodorai, 2016). While many of our observed effects were near 0.10, such “small” effects can still be theoretically meaningful, as they may accumulate over time to shape stable patterns of behavior (Funder and Ozer, 2019).

Second, although our ESEM measures temporally preceded the INT/EXT psychopathology measures, we cannot make inferences about the direction of causality. It is possible that higher levels of depression or externalizing in childhood predict lower levels of reward sensitivity or cognitive control in adolescence (Potsch and Rief, 2023). We focus on these analyses for the information they provide in terms of associations. These associations are complex, likely preceding the baseline timepoint and possibly reflecting bidirectional paths. Future work could attempt to establish causality and the direction of relationships more specifically, for example by leveraging more timepoints from this longitudinal study to estimate random intercept cross-lag panel models. The current study's results may inform such models by providing information about the structure of reward and control measures and the independence of their associations with psychopathology.

Third, because we were using existing data, we did not have control over what measures were included to tap reward and cognitive control. In particular, our battery may not have provided the best representation of reward sensitivity given the low reliability found for the individual differences measures from some of the reward sensitivity tasks we did not include (Supplementary Table S3). Additionally, two executive function tasks (Flanker and SST) showed a moderate test-retest reliability ~0.45 in our sample, but reliability is estimated to be higher (0.70–0.92, 0.72, respectively) with smaller test-retest windows (< 1 month; Karr et al., 2024; Zelazo et al., 2013; Soreni et al., 2009) compared to the large 2-year test-retest in the ABCD study. Given the developmental period, low 2-year test-retest correlations could reflect both unreliability as well as developmental change.

Fourth, we included age at baseline but not pubertal status as a covariate in our models. Previous research regarding the dual-systems model of adolescent risk-taking has suggested that changes in brain development due to pubertal development may be driving the imbalance between reward and cognitive control (Icenogle et al., 2017; Braams et al., 2015). Because pubertal status (as measured by self-report or hormonal analysis) has been determined to be an important factor in examinations of these constructs, we believe it is important to consider its effects beyond just controlling for it as a covariate. In particular, controlling for it as a covariate may remove variance of interest (e.g., variance driving the association between control and reward with psychopathology), making such models over-controlled (Streiner, 2016).

Finally, the absence of contextualizing how these findings change with the addition of socioeconomic status (SES) to the models should be considered. As has been shown in previous literature, the relationship between SES and psychopathology could be mediated by cognitive control/reward sensitivity (Duffy et al., 2023; Harden et al., 2017). As with pubertal status, we acknowledge that SES is multi-faceted and may have complex relationships to the development of psychopathology and associated risk factors (e.g., Deater-Deckard et al., 2019).

As is the case with all SEM, our model fit is based on how well it represents the underlying covariance matrix, but there are additional models that may be considered. An example would be allowing the mediated path from cognitive control to the Internalizing factor to be mediated by maladaptive emotional regulation (Kranzler et al., 2016). Likewise, the path from reward sensitivity to anhedonia could be mediated by low positive affect (Craske et al., 2016). Although an in-depth exploration of these relationships is beyond the scope of the current paper, such models are certainly worth examining in future work. Future work examining the longitudinal relationships between psychopathology and reward sensitivity is also of particular relevance, as it may be the case that the associations we observed may strengthen or change with age. Finally, the ABCD dataset includes genotyping information for participants which could enable future research to study the etiology of the associations observed here.

### 4.5 Conclusions and implications

Our findings highlight two key results. First, cognitive control and reward sensitivity appear multidimensional. Task-based and multiple survey-based cognitive control factors emerged. While we did not identify a unified task-based reward factor, the limited overlap between reward tasks and survey-based reward measures suggests these approaches capture separable aspects of reward sensitivity. Second, both cognitive control and reward sensitivity demonstrated unique associations with Internalizing and Externalizing psychopathology across multiple informants, even after accounting for shared rater variance. These findings underscore the critical importance of integrating task-based and survey measures alongside multi-informant perspectives to more fully characterize reward and control pathways as they relate to psychopathology risk in adolescence.

## Data Availability

Publicly available datasets were analyzed in this study. This data can be found here: https://10.15154/8873-zj65.
