# Supplementary material for: Multiple dimensions of reward-sensitivity and cognitive control relate to psychopathology: an exploratory structural equation modeling approach
Source: Front Psychol. 2026 Sep 11;17:1823262. doi: 10.3389/fpsyg.2026.1823262 (PMC13612339; doi:10.3389/fpsyg.2026.1823262)
Supplement: Supplementary file 1 [file Data_Sheet_1.pdf]

Supplementary Material

Multiple Dimensions of Reward-sensitivity and Cognitive Control Relate to Psychopathology: An Exploratory Structural Equation Modeling Approach

Analicia K. Howard[1,2], Roselinde H. Kaiser[2,3,4], Naomi P. Friedman[1,2,3]

[1]Institute for Behavioral Genetics, University of Colorado Boulder

[2]Department of Psychology and Neuroscience, University of Colorado Boulder

[3]Institute of Cognitive Science, University of Colorado Boulder

[4]Renée Crown Wellness Institute, University of Colorado Boulder

Supplemental Methods

A. Missingness Analysis

We examined whether the individuals who were missing psychopathology data at the year 2 follow-up differed significantly on demographics variables (self-reported race/ethnicity, parental educational attainment, parental household income) as well psychopathology status reported at baseline. There were no significant differences between those missing data at the year 2 follow-up and those not missing data at the year 2 follow-up on baseline psychopathology status (see Supplementary Table S5). The two groups (missing versus not missing at year 2 follow-up) differed in race/ethnicity, parental educational attainment, and parental household income, which is consistent with previous findings in ABCD (Ewing, et al., 2022).

B. Data Cleaning of Cognitive Control Tasks

For the *n*-back task we filtered by the ABCD-provided performance flag that drops anyone with overall task accuracy below 60%. We additionally filtered out anyone who appeared to not be engaging in the task as determined by missing all targets and correctly not answering for all non-targets (i.e. never responding). For the stop signal task, we filtered by the ABCD recommended "performflag," "violatorflag," and "glitchflag," wherein participants are filtered out for poor performance, for violating the race model assumptions (Verbruggen et al., 2019), and for a known glitch in which the stop signal response time was incorrectly recorded. More details about these filtering criteria are provided in the ABCD data release 5.0 notes.

C. Model Choice: Psychopathology Outcomes

We utilized a Multi-Trait Multi-Method (MTMM) approach where the "method" factors were for each rater, similar to what was done in the ESEM portion of the model. The trait

factors were two confirmatory factors comprised of internalizing and externalizing psychopathology outcomes.

Beyond a 2-factor CFA representing internalizing and externalizing disorders, we also tested a 3-factor CFA model with a 3rd "thought disorder" factor based on work by Caspi, et al. (2014); however, this model yielded no evidence for a distinct thought disorder factor in this sample. While the 2-factor CFA had acceptable fit, we wanted to ensure we were not missing any patterns in the data and thus we also examined 3-factor and 4-factor ESEMs (still including the confirmatory rater factors). The 3 and 4-factor ESEM of the psychopathology measures did not show clustering of items related to thought disorders (e.g., OCD or CBCL thought problems); these items instead clustered with internalizing or externalizing dimensions respectively. As such, the more parsimonious and interpretable 2-factor solution was chosen.

D. Replication Sample

Due to the large sample size of the ABCD study, it was possible to split the sample into two replication arms. A pipeline to do this in the ABCD sample has already been established. Full details can be found by reviewing the Fezco, et al., 2021 preprint on bioRxiv as well as the GitHub repository for the ABCD collection 3165 which represents the ABCD BIDS Community Collection (ABCC; https://github.com/ABCD-STUDY/nda-abcd-collection-3165). Of most interest are the ABCD Reproducible Matched Samples (ARMS), which were split into three demographically matched groups. ARMS 1 and 2 are to be used as independent datasets whereas ARM 3 is meant for model testing. The original first two ARMS were equal in sample size, however, due to updates to the pipeline (https://collection3165.readthedocs.io/en/stable/release_notes/) the current ARMS 1 and 2 samples have sample sizes of 5,739 and 5,734 respectively. The ARMS datasets were matched

on 9 demographics factors (research site, age, sex, race/ethnicity, grade level, highest level of education received by a parent, handedness, combined household income, and anesthesia exposure (due to the neuroimaging/neurodevelopmental component of the ABCD study). Because the ABCD study includes related individuals each ARMS dataset was also matched on family structure such that the same number of sibling/ pairs, and of triplets exists in each ARM dataset. A series of chi-squared tests were used to evaluate the amount of matching for each of these variables and showed no significant differences. Because the sample size differed slightly in the updated ARMS datasets, we confirmed that the current ARMS datasets also did not differ on any matched variables and found through a series of chi-squared tests that there were no statistically significant differences between the samples. The ARMS data can be downloaded from the National Data Archives website and can be found under ABCC collection 3165. Once downloaded the replication arms can be found by filtering on the “matched_group” variable.

E. Power Analysis

Post-hoc power was assessed with a Monte Carlo simulation in Mplus version 8.1, using the estimated factor loadings and sample size for the primary model with reward and control factors and psychopathology factors. We used these simulations to find the lowest ESEM-psychopathology factor correlation for which we had 80% power was achieved. While our lowest powered factor correlation had reached a minimum of 80% power at a correlation of 0.05, the majority of the factor correlations were still at 90% power or above at this level.

Table S1. Cognitive Control and Reward Sensitivity Survey Descriptive Statistics

Survey Item	Construct	Measure (Subscale)	Time point	Rater	Scoring	n	M	SD	Min	Max	Skew	Kurtosis
I feel excited and full of energy when I get something that I want	Reward Sensitivity	BAS (reward responsiveness)	BL	Youth	Likert 0-3	11854	2.08	0.94	0.00	3.00	-0.61	-0.73
When I am doing well at something, I like to keep doing this	Reward Sensitivity	BAS (reward responsiveness)	BL	Youth	Likert 0-3	11854	2.21	0.79	0.00	3.00	-0.75	-0.04
I get thrilled when good things happen to me	Reward Sensitivity	BAS (reward responsiveness)	BL	Youth	Likert 0-3	11853	2.19	0.83	0.00	3.00	-0.74	-0.18
It would excite me to win a contest	Reward Sensitivity	BAS (reward responsiveness)	BL	Youth	Likert 0-3	11853	2.38	0.78	0.00	3.00	-1.18	0.82
I get really excited when I see an opportunity to get something I like	Reward Sensitivity	BAS (reward responsiveness)	BL	Youth	Likert 0-3	11853	2.15	0.85	0.00	3.00	-0.64	-0.44
I often do things for no other reason than they might be fun	Reward Sensitivity	BAS (fun seeking)	BL	Youth	Likert 0-3	11853	1.09	0.98	0.00	3.00	0.53	-0.74
I am always willing to try something new, when I	Reward Sensitivity	BAS (fun seeking)	BL	Youth	Likert 0-3	11853	2.04	0.87	0.00	3.00	-0.51	-0.59

think it will be fun												
I crave excitement and new sensations	Reward Sensitivity	BAS (fun seeking)	BL	Youth	Likert 0-3	11853	1.49	0.97	0.00	3.00	0.1	-0.97
I often do things on the spur of the moment	Reward Sensitivity	BAS (fun seeking)	BL	Youth	Likert 0-3	11853	1.08	0.95	0.00	3.00	-0.51	-0.59
I enjoy taking risks	Reward Sensitivity	UPPS (sensation seeking)	BL	Youth	Likert 1-4	11853	2.45	1.05	1.00	4.00	-0.10	-1.21
I like new, thrilling things, even if they are a little scary	Reward Sensitivity	UPPS (sensation seeking)	BL	Youth	Likert 1-4	11853	3.05	0.95	1.00	4.00	-0.78	-0.29
I would like to learn to fly an airplane	Reward Sensitivity	UPPS (sensation seeking)	BL	Youth	Likert 1-4	11853	2.01	1.08	1.00	4.00	0.63	-0.97
I would like to ski very fast down a high mountain slope	Reward Sensitivity	UPPS (sensation seeking)	BL	Youth	Likert 1-4	11853	2.26	1.18	1.00	4.00	0.31	-1.41
Let’s pretend a kind person wanted to give you some money. Would you rather have $75 in three days or $115 in 3 months?	Reward Sensitivity	Cash Choice Questionnaire	BL	Youth	Likert 1-2	11876	1.63	0.52	1.00	2.00	-0.14	-1.17
When I am in a great mood, I tend to do things that can cause me problems	Cognitive Control	UPPS (positive urgency)	BL	Youth	Likert 1-4	11853	1.82	0.86	1.00	4.00	0.79	-0.17
I tend to act without	Cognitive Control	UPPS (positive urgency)	BL	Youth	Likert 1-4	11853	2.25	1.03	1.00	4.00	0.25	-1.11

thinking when I am very, very happy											
When I get really happy about something, I tend to do things that can lead to trouble	Cognitive Control	UPPS (positive urgency)	BL	Youth	Likert 1-4	11853	1.9	0.92	1.00	4.00	0.70	-0.47
I tend to lose control when I am in a great mood	Cognitive Control	UPPS (positive urgency)	BL	Youth	Likert 1-4	11853	2.02	1.01	1.00	4.00	0.58	-0.85
When I feel bad, I often do things I later regret in order to make myself feel better now	Cognitive Control	UPPS (negative urgency)	BL	Youth	Likert 1-4	11853	2.21	1.03	1.00	4.00	0.31	-1.10
When I am upset I often act without thinking	Cognitive Control	UPPS (negative urgency)	BL	Youth	Likert 1-4	11853	2.55	1.01	1.00	4.00	-0.13	-1.06
I like to stop and think about things before I do it	Cognitive Control	UPPS (lack of premeditation)	BL	Youth	Likert 1-4	11853	1.97	0.76	1.00	4.00	0.82	0.92
I try to take a careful approach to things	Cognitive Control	UPPS (lack of premeditation)	BL	Youth	Likert 1-4	11844	1.91	0.8	1.00	4.00	0.82	0.50
I am very careful	Cognitive Control	UPPS (lack of premeditation)	BL	Youth	Likert 1-4	11853	1.92	0.83	1.00	4.00	0.82	0.37
I tend to stop and think before doing things	Cognitive Control	UPPS (lack of premeditation)	BL	Youth	Likert 1-4	11853	1.94	0.82	1.00	4.00	0.8	0.42

I tend to get things done on time	Cognitive Control	UPPS (lack of perseverance)	BL	Youth	Likert 1-4	11853	1.82	0.84	1.00	4.00	0.92	0.31
Would be frightened by the thought of skiing fast down a steep slope	Reward Sensitivity	EATQ (high intensity pleasure)	Y2	Parent/ Caregiver	Likert 1-5	10331	3.19	1.33	1.00	5.00	-0.12	-1.15
Wouldn't be afraid to try a risky sport like deep sea diving	Reward Sensitivity	EATQ(high intensity pleasure)	Y2	Parent/ Caregiver	Likert 1-5	10331	2.91	1.28	1.00	5.00	0.03	-1.06
Would like driving a racing car	Reward Sensitivity	EATQ(high intensity pleasure)	Y2	Parent/ Caregiver	Likert 1-5	10331	3.05	1.34	1.00	5.00	-0.10	-1.16
Wouldn't want to go on the frightening rides at the fair	Reward Sensitivity	EATQ(high intensity pleasure)	Y2	Parent/ Caregiver	Likert 1-5	10331	3.56	1.32	1.00	5.00	-0.52	-0.90
Has a hard time finishing things on time	Cognitive Control	EATQ(activation control)	Y2	Parent/ Caregiver	Likert 1-5	10331	3.72	1.13	1.00	5.00	-0.48	-0.60
Usually finishes her/his homework before it's due	Cognitive Control	EATQ (activation control)	Y2	Parent/ Caregiver	Likert 1-5	10331	3.27	1.27	1.00	5.00	-0.26	-0.95
Usually gets started right away on difficult assignments	Cognitive Control	EATQ (activation control)	Y2	Parent/ Caregiver	Likert 1-5	10331	2.93	1.18	1.00	5.00	0.04	-0.82
Usually puts off working on a project until it is due	Cognitive Control	EATQ (activation control)	Y2	Parent/ Caregiver	Likert 1-5	10331	3.15	1.18	1.00	5.00	-0.09	-0.83
Has a hard time waiting his/her turn to speak when excited	Cognitive Control	EATQ (inhibitory control)	Y2	Parent/ Caregiver	Likert 1-5	10332	3.18	1.19	1.00	5.00	-0.05	-0.87

Opens presents before s/he is supposed to	Cognitive Control	EATQ (inhibitory control)	Y2	Parent/ Caregiver	Likert 1-5	10333	4.41	0.9	1.00	5.00	-1.59	2.11
Is more likely to do something s/he shouldn't do the more s/he tries to stop her/himself	Cognitive Control	EATQ (inhibitory control)	Y2	Parent/ Caregiver	Likert 1-5	10331	4.24	0.91	1.00	5.00	-1.13	0.83
Is able to stop him/herself from laughing at inappropriate times	Cognitive Control	EATQ (inhibitory control)	Y2	Parent/ Caregiver	Likert 1-5	10331	3.38	1.14	1.00	5.00	-0.47	-0.46
Is usually able to stick with his/her plans and goals	Cognitive Control	EATQ (inhibitory control)	Y2	Parent/ Caregiver	Likert 1-5	10331	3.57	0.92	1.00	5.00	-0.49	0.23
Is often in the middle of doing one thing and then goes off to do something else without finishing it	Cognitive Control	EATQ (attention)	Y2	Parent/ Caregiver	Likert 1-5	10331	3.43	1.02	1.00	5.00	-0.24	-0.35

Note. UPPS= Urgency, Premeditation, Perseverance, and Sensation-Seeking; Impulsive Behavior Scale; BAS= Behavioral Activation Scale; EATQ= Early Adolescent Temperament Questionnaire; BL= Baseline; Y2= Year 2 follow-up. Italics denote reverse-coded items.

Table S2. Items from Survey Subscales Not Included in Analyses

Survey Item	Measure (Subscale)
Sometimes when I feel bad, I keep doing something even though it is making me feel worse	UPPS (Negative Urgency)
When I feel rejected, I often say things that I later regret	UPPS (Negative Urgency)
Finds it easy to really concentrate on a problem	EATQ Attention
When interrupted or distracted, forgets what s/he was about to say	EATQ Attention
Is good at keeping track of several different things	EATQ Attention
Pays close attention when someone tells her/him how to do something	EATQ Attention
Has a difficult time tuning out background noise and concentrating when trying to study	EATQ Attention
Thinks travelling to Africa or India would be exciting and fun	EATQ High Intensity Pleasure
Expresses a desire to travel to exotic places when s/he hears about them	EATQ High Intensity Pleasure
Is energized by being in large crowds of people	EATQ High Intensity Pleasure

Note. UPPS= Urgency, Premeditation, Perseverance, and Sensation Seeking; EATQ= Early Adolescent Temperament Questionnaire. We did not include items from the Behavioral Activation Drive Subscale.

Table S3. Task Reliabilities

Task (dependent measure)	Hypothesized Construct	Reliability	Used in analysis
Game of Dice (difference score of number of safe choices - number of risky choices)	Reward Sensitivity	Split-half: 0.92	yes
Delay Discounting (discounting constant, k)	Reward Sensitivity	Split-half: 0.74	yes
Monetary Incentive Delay (difference score of mean reaction time of all reward trials - all neutral trials)	Reward sensitivity	Split-half: 0.15	no
Monetary Incentive Delay (difference score of mean reaction time of all loss - all reward trials)	Reward Sensitivity	Split-half: 0.14	no
Emotional nBack (2 back accuracy)	Cognitive Control	Split-half: 0.73	yes
Stop Signal Task (stop signal reaction time)	Cognitive Control	Test-retest:0.47	yes
List Sorting Working Memory	Cognitive Control	Test-retest: 0.86[a]	yes
Flanker Inhibitory Control and Attention (reaction time and accuracy)	Cognitive Control	Test-retest: 0.44	yes
Dimensional Change Card Sort (reaction time and accuracy)	Cognitive Control	Test-retest: 0.92[b]	yes

Note. Reliability was estimated within this sample unless otherwise noted.
[a] Reliability measured in sample described in Tulsky, et al., 2013.
[b] Reliability measured in sample described in Zelazo, et al., 2013.

Table S4. Task Descriptive Statistics

Task	Description of Measure	Timepoint	n	M	SD	Min	Max	Skew	Kurtosis	Scoring
Flanker	Measures interference control and attention. Participants indicate which direction a central arrow is pointing while ignoring flanking arrows that may be pointing in the same or opposite direction (Luciana, et al., 2018).	BL	11723	94.00	9.14	51	116	-1.00	1.49	Reaction Time (Toolbox scored)
Stop Signal Task (SST)	This task was administered during an fMRI scan and measures response inhibition. Participants are indicate the direction of an arrow (left or right) but make no response when the arrow turns to point up on 16.67% of trials (Casey, et al., 2018).	BL	6717	293.71	58.75	82.78	556.91	0.20	0.33	Stop Signal Reaction Time
Emotional n-back	This task was administered during an fMRI scan and measures working memory as well as emotional reactivity. Participants are instructed to match stimuli based on a previously seen target that is either 2 back or 0 trials back (Casey, et al., 2018).	BL	9350	0.79	0.09	0.61	1.00	-0.04	1.20	2-back Accuracy
List Sorting Working Memory	Measures working memory. Participants are shown and name aloud pictures of animals and foods of different sizes. Participants must repeat the list back, but in size order. List sort begins with only a single category but increase up to seven depending on accuracy of previous trials (Tulsky, et al., 2013; Luciana, et al., 2018).	BL	11680	96.65	12.08	36	136	-0.54	0.87	Number of Correct Responses (Toolbox scored)
Dimensional Change Card Sort	Measures cognitive flexibility. Participants sort objects to match two objects shown at the bottom of the screen based on directions to match via color or shape, which switch from trial to trial (Zelazo, et al., 2013, 2014; Luciana, et al., 2018).	BL	11724	92.52	9.51	50	120	-0.82	2.04	Reaction Time (Toolbox scored)
Game of Dice	Participants guess what number on a die will be thrown. They choose between combinations of one, two, three, or four numbers. Choosing one or two numbers is considered a “risky” choice	Y2	9527	2.08	10.14	-18	18	-0.26	-0.88	#safe choices-#risky choices

	whereas choosing a combination of three or four numbers is considered a "safe" choice (Pacheco-Colón, et al., 2021; Brand et al., 2005)									
Delay Discounting	Participants choose between hypothetical smaller immediate rewards or a larger ($100) delayed reward. There were 7 delays ranging from 6 hours to 5 years. The task adjusts the immediate reward up or down depending on the participant's last decision until an indifference point is reached in which the immediate reward is equal to value of the larger reward (Koffarnus & Bickel, 2014; Luciana et al., 2018). The indifference points form a hyperbolic curve in which the area under the curve (AUC) can be estimated. The steepness of this curve is characterized by the discounting constant k, which is typically log transformed to reduce skew (Bickel, et al., 2014).	Y1	8619	-1.95	3.71	-8.74	9.48	0.29	0.37	ln(k)

Note. BL= Baseline; Y2= Year 2 follow-up; Y1= Year 1 follow-up; M= mean; SD= Standard Deviation; Min= Minimum; Max= Maximum.

Table S5. Missing Data Analysis

Independent Variable	Dependent Variable	Beta	Standard Error	*p*-value	Odds Ratio	Explanation
CBCL BL Summary Score (Continuous, Standardized)	Y2 CBCL Missing (Yes/No)	0.04	0.03	0.21	1.04	Missing Y2 CBCL scores is not related to BL scores.
KSADS BL Diagnosis (Contrast-coded: +0.5 for positive diagnosis, -0.5 for negative diagnosis**)**	Y2 KSADS Missing (Yes/No)	0.23	0.14	0.09	1.27	Missing Y2 KSADS diagnoses is not related to BL diagnoses.
BPMT BL Summary Score (Continuous, Standardized)	Y2 BPMT Missing (Yes/No)	0.20	0.03	<0.0001	1.22	Missing Y2 BPMT diagnosis is significantly related to BL diagnosis.
Categorical Race/Ethnicity (0= White, 1= Not white)	Y2 CBCL Missing (Yes/No)	0.75	0.07	<0.0001	2.12	342/6172 people are white identifying and missing CBCL data at Y2 (5.54%). 629/5694 of people identify as another race/ethnicity besides white and are missing CBCL data at Y2 (11.04%). 52% of full sample at Y2 are white identifying.
Categorical Race/Ethnicity (0= White, 1= Not white)	Y2 KSADS Missing (Yes/No)	0.71	0.07	<0.0001	2.02	52% of full sample at Y2 are white identifying. 370/6172 are white identifying and missing KSADS data at Y2 (5.99%). 652/5694 people identify as something other than white (11.45%).
Categorical Race/Ethnicity (0= White, 1= Not white)	Y2 BPMT Missing (Yes/No)	0.77	0.04	<0.0001	2.16	52% of full sample are white identifying. 3451/6172 (55.91%) are white identifying and missing BPMT data at Y2. 4169/5694 (73.21%) identify as a race/ethnicity other than white and are missing BPMT data at Y2.
Parent Education BL (Continuous, Standardized)	Y2 CBCL Missing (Yes/No)	-0.38	0.03	<0.0001	0.68	Average education for full sample is Associate's Degree- occupational; Average education for those missing CBCL data at Y2 is Associate's Degree- educational
Parent Education BL (Continuous, Standardized)	Y2 KSADS Missing (Yes/No)	-0.36	0.03	<0.0001	0.69	Average education for full sample is a Master's Degree; Average education for those missing KSADS at Y2 is some college- associate's degree.

Parent Education BL (Continuous, Standardized)	Y2 BPMT Missing (Yes/No)	-0.11	0.008	<0.0001	0.9	Average education for the full sample is Associate's degree- education. The average education for those missing BPMT data at Y2 is Associate's degree- occupational.
Household Income BL (Continuous, Standardized)	Y2 CBCL Missing (Yes/No)	-0.41	0.03	<0.0001	0.66	Average household income for full sample is between 50-75 thousand dollars/year. The Average household income for those missing CBCL data at Y2 is 35-50 thousand dollars/year.
Household Income BL (Continuous, Standardized)	Y2 KSADS Missing (Yes/No)	-0.38	0.03	<0.0001	0.68	Average household income for full sample is between 50-75 thousand dollars/year. The Average household income for those missing KSADS data at Y2 is 35-50 thousand dollars/year.
Household Income BL (Continuous, Standardized)	Y2 BPMT Missing (Yes/No)	-0.31	0.02	<0.0001	0.73	Average household income for full sample is 50-75 thousand dollars/year. The average household income for those missing BPMT data at Y2 is 50-75 thousand dollars/year

Note. CBCL= Child Behavior Checklist; KSADS= Kiddie Schedule of Affective Disorders and Schizophrenia; BPMT= Brief Problem Monitor Teacher Report; BL= Baseline; Y2= year 2 follow-up.

Table S6. List of KSADS-5 Diagnoses and Diagnostic Classes

Disorder Class	KSADS-5 Diagnosis
Depressive Disorders	Persistent Depressive Disorder (Dysthymia) - Present
	Persistent Depressive Disorder (Dysthymia) - In Partial Remission
	Major Depressive Disorder - Present
	Major Depressive Disorder - In Partial Remission
	Unspecified Depressive Disorder - Current
	Persistent Depressive Disorder (Dysthymia) - Past
	Major Depressive Disorder - Past
	Unspecified Depressive Disorder - Past
	Persistent Depressive Disorder (Dysthymia) - Present
	Persistent Depressive Disorder (Dysthymia) - In Partial Remission
	Major Depressive Disorder - Present
	Major Depressive Disorder - In Partial Remission
	Unspecified Depressive Disorder - Current
	Persistent Depressive Disorder (Dysthymia) - Past
	Major Depressive Disorder - Past
	Unspecified Depressive Disorder - Past
Bipolar Disorders	Bipolar II, Most Recent Past Hypomanic
	Bipolar II, Currently Hypomanic
	Bipolar II, Currently Depressed
	Bipolar I, Current Episode Depressed
	Bipolar I, Currently Hypomanic
	Bipolar I, Current Episode Manic
	Bipolar I, Most Recent Past Episode Manic
	Bipolar I, Most Recent Past Episode Depressed
	Unspecified Bipolar and Related Disorder - Current
	Unspecified Bipolar and Related Disorder - Past
	Bipolar II, Most Recent Past Hypomanic
	Bipolar II, Currently Hypomanic
	Bipolar II, Currently Depressed
	Bipolar I, Current Episode Depressed
	Bipolar I, Currently Hypomanic

	Bipolar I, Current Episode Manic<br>Bipolar I, Most Recent Past Episode Manic<br>Bipolar I, Most Recent Past Episode Depressed<br>Unspecified Bipolar and Related Disorder - Current<br>Unspecified Bipolar and Related Disorder - Past
Anxiety Disorders	Social Anxiety Disorder - Present<br>Separation Anxiety Disorder - Present*<br>Generalized Anxiety Disorder - Present<br>Social Anxiety Disorder - Past<br>Separation Anxiety Disorder - Past*<br>Generalized Anxiety Disorder - Past
Disruptive Behavior Disorders	Oppositional Defiant Disorder - Present*<br>Oppositional Defiant Disorder - Past*<br>Conduct Disorder, Childhood Onset - Present<br>Conduct Disorder, Adolescent Onset - Present<br>Conduct Disorder, Childhood Onset - Past<br>Conduct Disorder, Adolescent Onset - Past
Attention Deficit and Hyperactivity Disorder	Attention Deficit and Hyperactivity Disorder- Present*<br>Attention Deficit and Hyperactivity Disorder- Past*<br>Attention Deficit and Hyperactivity Disorder- Partial Remission*<br>Unspecified Attention Deficit and Hyperactivity Disorder*

* only administered to parent/caregiver.

Table S7. Frequencies of KSADS-5 Diagnoses

KSADS-5 Diagnostic Class	Timepoint	Rater	Positive Diagnosis *n*	Negative Diagnosis *n*	Total *n*	Percent with positive diagnosis
Depressive Disorders	Y2	Youth	635	10224	10859	5.84
Depressive Disorders	Y2	Parent	835	9895	10730	7.78
Bipolar Disorders	Y2	Youth	417	10442	10859	3.84
Bipolar Disorders	Y2	Parent	274	10456	10730	2.55
Anxiety Disorders	Y2	Youth	509	10350	10859	4.69
Anxiety Disorders	Y2	Parent	1663	9067	10730	15.50
Disruptive Behavior Disorders	Y2	Youth	121	10738	10859	1.14
Disruptive Behavior Disorders	Y2	Parent	1484	9246	10730	13.83
Obsessive Compulsive Disorder*	Y2	Parent	944	9786	10730	8.80
Attention Deficit/Hyperactivity Disorder*	Y2	Parent	1426	9324	10750	13.27

Note: Y2= Year 2 follow-up.
*only administered to parent/caregiver.

Table S8. Descriptive Statistics for Psychopathology Measures

Psychopathology Measure	Timepoint	Rater	*n*	M	SD	Min	Max	Skew	Kurtosis
CBCL: Internalizing	Y2	Parent	10896	4.94	5.66	0	50	2.06	5.75
CBCL: Externalizing	Y2	Parent	10896	3.92	5.58	0	50	2.53	8.54
CBCL: Attention Problems	Y2	Parent	10896	2.73	3.33	0	19	1.52	2.14
CBCL: Thought Problems	Y2	Parent	10896	1.44	2.11	0	22	2.44	8.43
CBCL Social Problems	Y2	Parent	10896	1.32	2.08	0	17	2.41	7.29
Brief Problem Monitor-Youth: Internalizing	Y2	Parent	10616	1.84	2.24	0	12	1.63	2.71
Brief Problem Monitor-Youth: Externalizing	Y2	Parent	10616	2.09	2.02	0	13	1.16	1.48
Brief Problem Monitor-Youth: Attention Problems	Y2	Parent	10616	3.37	2.73	0	12	0.63	-0.33
Brief Problem Monitor-Teacher: Internalizing	Y2	Parent	4246	1.58	2.16	0	12	1.73	2.96
Brief Problem Monitor-Teacher: Externalizing	Y2	Parent	4246	0.88	1.89	0	12	2.79	8.40
Brief Problem Monitor-Teacher: Attention Problems	Y2	Parent	4246	2.37	3.00	0	12	1.26	0.71

Note. CBCL= Child Behavior Checklist; Y2= Year 2 follow-up; M= mean; SD= Standard Deviation; Min= Minimum; Max= Maximum.

Table S9. Standardized Regression Coefficients for Effects of Covariates on Psychopathology Measures

Psychopathology Measure	Sex		Age at Baseline	
	β	*p*	β	*p*
CBCL Social Problems	-0.012	0.120	-0.006	0.452
CBCL Social Problems	0.010	0.253	-0.008	0.414
CBCL Thought Problems	-0.035	<0.001	0.026	0.001
CBCL Thought Problems	-0.019	0.048	0.013	0.181
CBCL Attention Problems	-0.070	<0.001	0.002	0.771
CBCL Attention Problems	-0.064	<0.001	-0.003	0.793
CBCL Internalizing	0.0070	0.290	0.054	<0.001
CBCL Internalizing	0.026	0.003	0.037	<0.001
CBCL Externalizing	-0.031	<0.001	0.003	0.697
CBCL Externalizing	-0.015	0.065	0.008	0.373
BPM Youth Internalizing	-0.015	0.075	-0.008	0.357
BPM Youth Internalizing	0.053	<0.001	0.013	0.156
BPM Youth Externalizing	-0.039	<0.001	0.000	0.956
BPM Youth Externalizing	0.007	0.423	0.013	0.156
BPM Youth Attention	-0.053	<0.001	-0.016	0.048
BPM Youth Attention	0.002	0.830	-0.010	0.266
BPM Teacher Internalizing	-0.009	0.508	0.015	0.262
BPM Teacher Internalizing	0.011	0.462	-0.001	0.956
BPM Teacher Externalizing	-0.058	<0.001	-0.004	0.757
BPM Teacher Externalizing	-0.046	<0.001	-0.015	0.318
BPM Teacher Attention	-0.090	<0.001	-0.003	0.841
BPM Teacher Attention	-0.079	<0.001	-0.010	0.509
KSADS-5 Depressive Disorders (Youth)	0.005	0.800	0.038	0.048
KSADS-5 Depressive Disorders (Youth)	0.033	0.102	0.037	0.061
KSADS-5 Anxiety Disorders (Youth)	-0.009	0.659	0.053	0.012
KSADS-5 Anxiety Disorders (Youth)	0.064	0.001	0.066	<0.001
KSADS-5 Bipolar Disorders (Youth)	-0.040	0.013	0.009	0.637
KSADS-5 Bipolar Disorders (Youth)	0.009	0.691	-0.034	0.161
KSADS-5 Disruptive Disorders (Youth)	0.025	0.434	0.007	0.834
KSADS-5 Depressive Disorders (Parent)	-0.028	0.113	0.050	0.015

KSADS-5 Depressive Disorders (Parent)	-0.004	0.839	0.049	0.010
KSADS-5 Anxiety Disorders (Parent)	0.005	0.673	0.018	0.137
KSADS-5 Anxiety Disorders (Parent)	0.024	0.109	0.030	0.036
KSADS-5 Bipolar Disorders (Parent)	-0.011	0.560	0.009	0.702
KSADS-5 Bipolar Disorders (Parent)	-0.017	0.482	0.034	0.210
KSADS-5 Obsessive Compulsive Disorder (Parent)	-0.066	<0.001	0.035	0.011
KSADS-5 Obsessive Compulsive Disorder (Parent)	-0.016	0.337	0.059	0.001
KSADS-5 Disruptive Disorders (Parent)	-0.047	<0.001	0.008	0.534
KSADS-5 Disruptive Disorders (Parent)	-0.028	0.052	0.024	0.102

Note. CBCL= Childhood Behavior Checklist; KSADS= Kiddie Schedule of Affective Disorders and Schizophrenia.

Table S10. Fit Statistics for Full 1-Factor Through 9-Factor Exploratory Structural Equation Models with Covariates, Method/Rater Factors, and Psychopathology

	Fit Statistics					
Number of Factors	χ^2 (df)	CFI	RMSEA	SRMR	R^2 INT	R^2 EXT
1	104178.54 (2399)	0.643	0.060	0.073	0.195	0.683
2	68690.74 (2348)	0.767	0.049	0.060	0.198	0.703
3	50847.95 (2298)	0.829	0.042	0.052	0.255	0.745
4	39356.89 (2249)	0.870	0.037	0.046	0.244	0.762
5	31319.11 (2201)	0.898	0.033	0.042	0.307	0.761
6	24051.52 (2154)	0.923	0.029	0.038	0.310	0.793
**7**	**19470.99 (2108)**	**0.939**	**0.026**	**0.036**	**0.344**	**0.878**
8	17256.69 (2063)	0.947	0.025	0.034	0.349	0.867
9	15964.99 (2019)	0.951	0.024	0.033	0.380	0.926

Note. Boldface type indicates selected models. All χ^2 $p<.001$. χ^2= Chi-Square; CFI= Comparative Fit Index; RMSEA= Root Mean Square Error of Approximation; SRMR= Standardized Root Mean Square Residual; R^2 indicates proportion of variance explained; INT= Internalizing factor; EXT= Externalizing factor.

Table S11. 4-factor ESEM Without Psychopathology

		Factor Loadings			
Indicator	Instrument/ Timepoint	Factor 1	Factor 2	Factor 3	Factor 4
I feel excited and full of energy when I get something that I want(bisbas8)	BAS BL	**0.56**	0.01	-0.12	0.10
When I am doing well at something, I like to keep doing this(bisbas9)	BAS BL	**0.59**	-0.12	0.01	-0.08
I get thrilled when good things happen to me (bis bas 10)	BAS BL	**0.66**	-0.09	-0.06	-0.05
It would excite me to win a contest(bisbas11)	BAS BL	**0.61**	-0.03	-0.04	-0.08
I get really excited when I see an opportunity to get something I like(bisbas12)	BAS BL	**0.65**	-0.03	-0.11	0.03
I often do things for no other reason than they might be fun(bisbas17)	BAS BL	**0.39**	0.15	-0.05	0.26
I am always willing to try something new, when I think it will be fun(bisbas19)	BAS BL	**0.57**	-0.02	0.02	0.04
I crave excitement and new sensations(bisbas18)	BAS BL	**0.59**	0.07	0.02	0.04
Cash Choice	Cash Choice	0.06	0.02	-0.02	0.01
Opens presents before s/he is supposed to (eatq_open_present)	EATQ Y2	-0.03	0.15	0.22	-0.21
Would be frightened by the thought of skiing fast down a steep slope (eatq_ski_slope)	EATQ Y2	0.04	0.21	**0.43**	0.02
Usually does something fun for awhile before starting her/his homework, even though s/he is not supposed to (eatq_before_hw)	EATQ Y2	-0.07	-0.07	**0.45**	0.03

Wouldn't be afraid to try a risky sport like deep sea diving (eatq_sea_dive)	EATQ Y2	0.11	0.29	**0.39**	-0.02
Would like driving a racing car (eatq_race_car)	EATQ Y2	0.12	0.25	0.22	0.00
Wouldn't want to go on the frightening rides at the fair (eatq_rides_scared)	EATQ Y2	0.08	0.19	**0.39**	0.01
I enjoy taking risks (upps12)	UPPS BL	0.21	**0.34**	0.08	0.09
I like new, thrilling things, even if they are a little scary(upps18)	UPPS BL	**0.33**	0.23	0.08	0.05
I would like to learn to fly an airplane(upps21)	UPPS BL	0.11	-0.07	0.05	0.07
I would like to ski very fast down a high mountain slope (upps27)	UPPS BL	0.21	0.20	0.12	0.03
I often do things on the spur of the moment(bisbas20)	UPPS BL	**0.41**	0.18	-0.02	0.20
Has a hard time finishing things on time (eatq_finish)	EATQ Y2	-0.02	0.01	**0.68**	-0.05
Has a hard time waiting his/her turn to speak when excited (eatq_turn_taking)	EATQ Y2	-0.07	-0.06	0.24	-0.02
When asked to do something does it right away, even if s/he doesn't want to (eatq_right_away)	EATQ Y2	-0.02	-0.06	**0.44**	0.02
Is more likely to do something s/he shouldn't do the more s/he tries to stop her/himself (eatq_impulse)	EATQ Y2	-0.01	0.11	**0.31**	-0.18
Usually finishes her/his homework before it's due (eatq_finish_hw)	EATQ Y2	-0.03	-0.03	**0.66**	-0.01
Usually gets started right away on difficult assignments (eatq_early_start)	EATQ Y2	-0.04	-0.07	**0.76**	0.06

Usually puts off working on a project until it is due (eatq_puts_off)	EATQ Y2	-0.05	-0.10	**0.70**	0.09
Is able to stop him/herself from lauging at inappropriate times (eatq_laugh_control)	EATQ Y2	0.01	0.14	0.06	-0.17
Is often in the middle of doing one thing and then goes off to do something else without finishing it (eatq_sidetracked)	EATQ Y2	-0.04	0.00	**0.54**	-0.04
Is usually able to stick with his/her plans and goals (eatq_stick_to_plan)	EATQ Y2	-0.02	0.06	**0.49**	-0.10
I like to stop and think about things before I do it (upps6)	UPPS BL	-0.22	**0.59**	-0.18	0.04
When I feel bad, I often do things I later regret in order to make myself feel better now (upps7)	UPPS BL	0.10	-0.09	0.01	**0.37**
I try to take a careful approach to things(upps16)	UPPS BL	-0.23	**0.47**	-0.11	0.01
When I am upset I often act without thinking(upps17)	UPPS BL	0.07	0.25	-0.05	0.29
I tend to get things done on time(upps19)	UPPS BL	-0.18	0.27	**-0.32**	0.08
I am very careful (upps23)	UPPS BL	-0.14	**0.58**	-0.12	0.01
I tend to stop and think before doing things (upps28)	UPPS BL	-0.22	**0.71**	-0.15	0.02
When I am in a great mood, I tend to do things that can cause me problems (upps35)	UPPS BL	-0.04	0.15	0.06	**0.63**
I tend to act without thinking when I am very, very happy (upps36)	UPPS BL	0.09	0.02	0.03	**0.69**
When I get really happy about something, I tend to do things that can lead to trouble (upps37)	UPPS BL	0.00	0.19	0.05	**0.73**

I tend to lose control when I am in a great mood (upps39)	UPPS BL	0.09	0.04	0.02	**0.69**
Game of Dice	Task Y2	-0.01	0.08	0.06	-0.14
Delay Discounting- ln(k)	Task Y1	-0.01	-0.10	-0.03	0.14
Flanker	Task BL	0.08	**0.32**	0.09	-0.31
List Sort	Task BL	0.02	**0.32**	0.13	**-0.34**
Card Sort	Task BL	0.06	**0.31**	0.15	**-0.35**
Nback	Task BL	0.03	0.29	0.12	**-0.30**
SST	Task BL	-0.03	-0.08	-0.07	0.15
Factor Correlations					
F1		1			
F2		–0.01	1		
F3		0.02	–0.07	1	
F4		0.12**	0.27**	–0.21**	1

Note. BAS= Behavioral Activation Scale; UPPS= Urgency, Premeditation, Perseverance, Sensation-Seeking; EATQ= Early Adolescent Temperament Questionnaire; EF= Executive Function; BL= Baseline; Y2= Year 2 Follow-up; *denotes p<0.01; **denotes p<0.001. Italics denotes a reverse-coded item.

Table S12. 7-factor Final Model ESEM with Psychopathology Solution for Replication Arm 1

Item	Instrument Timepoint	F1	F2	F3	F4	F5	F6	F7	Internalizing Indicator	INT	Externalizing Indicator	EXT
I feel excited and full of energy when I get something that I want(bisbas8)	BAS BL	**0.61**	-0.06	0.11	0.05	-0.03	-0.09	0.03	Y_DD_A_2	**0.49**	Y_DSRD_A_2	**0.42**
When I am doing well at something, I like to keep doing this(bisbas9)	BAS BL	**0.55**	0.02	-0.16	-0.10	0.01	0.01	0.01	Y_AD_A_2	**0.47**	ADHD_A_2	**0.71**
I get thrilled when good things happen to me (bis bas 10)	BAS BL	**0.66**	0.01	-0.05	-0.05	0.03	-0.10	-0.01	DD_A_2	**0.50**	DSRD_A_2	**0.57**
It would excite me to win a contest(bisbas11)	BAS BL	**0.60**	0.01	-0.05	-0.09	-0.04	0.04	0.06	AD_A_2	**0.46**	CBCL_ATT	**0.73**
I get really excited when I see an opportunity to get something I like(bisbas12)	BAS BL	**0.68**	-0.08	0.05	-0.02	-0.05	-0.05	0.04	CBCL_INT	**0.67**	CBCL_EXT	**0.61**
I often do things for no other reason than they might be fun(bisbas17)	BAS BL	**0.38**	-0.02	0.07	0.29	-0.02	0.09	-0.02	BPMY_INT	**0.41**	BPMY_ATT	**0.46**
I am always willing to try something new, when I think it will be fun(bisbas19)	BAS BL	**0.48**	-0.02	-0.15	0.05	0.01	0.19	-0.02	BPMT_INT	**0.47**	BPMY_EXT	**0.36**
I crave excitement and new sensations(bisbas18)	BAS BL	**0.52**	0.04	-0.10	0.09	0.02	0.18	-0.06	OCD_A_2	**0.41**	BPMT_ATT	**0.56**

Cash Choice	Cash Choice Task	0.10	-0.03	0.05	-0.01	0.04	0.01	-0.05	BPMT_EXT	**0.46**
Opens presents before s/he is supposed to (eatq_open_present)	EATQ Y2	-0.02	0.20	0.00	-0.05	-0.04	0.04	**0.46**	Y_BD_A_2	0.26
Would be frightened by the thought of skiing fast down a steep slope (eatq_ski_slope)	EATQ Y2	-0.02	-0.06	0.03	-0.03	0.00	**0.50**	**0.33**	BD_A_2	**0.41**
Usually does something fun for awhile before starting her/his homework, even though s/he is not supposed to (eatq_before_hw)	EATQ Y2	-0.02	-0.08	0.01	-0.01	**0.41**	-0.05	0.28	CBCL_THT	**0.46**
Wouldn't be afraid to try a risky sport like deep sea diving (eatq_sea_dive)	EATQ Y2	-0.02	0.00	0.05	-0.05	0.11	**0.63**	0.06	CBCL_SOC	**0.49**
Would like driving a racing car (eatq_race_car)	EATQ Y2	-0.01	-0.02	0.01	-0.03	-0.01	**0.54**	-0.03		
Wouldn't want to go on the frightening rides at the fair (eatq_rides_scared)	EATQ Y2	0.05	-0.04	0.05	-0.03	0.04	**0.43**	0.26		
I enjoy taking risks (upps12)	UPPS BL	0.11	0.15	0.07	0.18	0.00	**0.31**	-0.02		
I like new, thrilling things, even if they	UPPS BL	0.19	0.11	-0.05	0.14	-0.01	**0.32**	-0.04		

are a little scary(upps18)								
I would like to learn to fly an airplane(upps21)	UPPS BL	-0.08	-0.07	-0.29	0.03	-0.09	**0.31**	-0.01
I would like to ski very fast down a high mountain slope (upps27)	UPPS BL	0.02	0.07	-0.12	0.05	-0.05	**0.48**	-0.02
I often do things on the spur of the moment(bisbas20)	BAS BL	**0.38**	0.01	0.06	0.24	0.03	0.16	-0.05
Has a hard time finishing things on time (eatq_finish)	EATQ Y2	0.02	0.08	-0.02	0.03	**0.51**	-0.01	**0.43**
Has a hard time waiting his/her turn to speak when excited (eatq_turn_taking)	EATQ Y2	-0.07	-0.01	-0.13	0.05	-0.02	-0.01	**0.57**
When asked to do something does it right away, even if s/he doesn't want to (eatq_right_away)	EATQ Y2	-0.04	-0.05	-0.09	0.03	**0.41**	0.05	0.22
Is more likely to do something s/he shouldn't do the more s/he tries to stop her/himself (eatq_impulse)	EATQ Y2	0.02	0.14	-0.01	-0.04	0.06	0.01	**0.52**
Usually finishes her/his homework before it's due (eatq_finish_hw)	EATQ Y2	-0.01	0.06	-0.01	-0.03	**0.77**	0.00	-0.07

Usually gets started right away on difficult assignments (eatq_early_start)	EATQ Y2	-0.02	-0.03	0.00	0.00	**0.90**	0.02	-0.06
Usually puts off working on a project until it is due (eatq_puts_off)	EATQ Y2	0.03	-0.08	0.03	0.01	**0.70**	-0.03	0.19
Is able to stop him/herself from lauging at inappropriate times (eatq_laugh_control)	EATQ Y2	-0.04	0.17	-0.02	-0.06	0.07	0.07	0.08
Is often in the middle of doing one thing and then goes off to do something else without finishing it (eatq_sidetracked)	EATQ Y2	0.03	0.03	0.03	0.01	**0.37**	-0.04	**0.48**
Is usually able to stick with his/her plans and goals (eatq_stick_to_plan)	EATQ Y2	-0.05	0.12	-0.06	-0.02	**0.46**	0.08	0.13
I like to stop and think about things before I do it (upps6)	UPPS BL	0.01	-0.05	**0.72**	0.00	-0.02	0.00	-0.02
When I feel bad, I often do things I later regret in order to make myself feel better now (upps7)	UPPS BL	0.04	-0.07	-0.12	**0.37**	-0.03	-0.03	0.03
I try to take a careful approach to things(upps16)	UPPS BL	-0.12	-0.03	**0.51**	-0.01	-0.06	0.12	0.03

When I am upset I often act without thinking(upps17)	UPPS BL	0.09	0.11	0.19	**0.38**	-0.01	-0.09	-0.06
I tend to get things done on time(upps19)	UPPS BL	-0.10	-0.07	**0.36**	0.04	-0.22	-0.03	-0.01
I am very careful (upps23)	UPPS BL	-0.04	0.07	**0.59**	0.04	-0.01	0.12	-0.02
I tend to stop and think before doing things (upps28)	UPPS BL	0.00	0.04	**0.82**	0.03	0.01	0.00	0.01
When I am in a great mood, I tend to do things that can cause me problems (upps35)	UPPS BL	-0.14	0.02	0.01	**0.68**	0.00	0.03	-0.02
I tend to act without thinking when I am very, very happy (upps36)	UPPS BL	0.02	-0.10	-0.04	**0.69**	-0.01	-0.01	0.04
When I get really happy about something, I tend to do things that can lead to trouble (upps37)	UPPS BL	-0.07	0.03	0.06	**0.80**	0.00	0.02	0.01
I tend to lose control when I am in a great mood (upps39)	UPPS BL	0.03	-0.11	-0.03	**0.69**	0.00	-0.03	0.02
Game of Dice	Task Y2	-0.02	0.17	0.01	-0.03	0.04	-0.04	-0.01
Delay Discounting-ln(k)	Task Y1	0.00	-0.13	-0.03	0.09	-0.02	-0.06	0.06
Flanker	Task BL	0.01	**0.59**	0.02	0.01	-0.02	-0.02	0.01
List Sort	Task BL	-0.02	**0.54**	0.05	-0.04	0.03	0.02	0.02
Card Sort	Task BL	0.00	**0.63**	-0.01	0.00	0.03	-0.03	0.00

Nback	Task BL	-0.04	**0.52**	0.00	0.00	-0.01	-0.01	0.03
SST	Task BL	-0.03	-0.25	0.07	-0.01	0.03	0.07	-0.05

Factor Correlations	F1	F2	F3	F4	F5	F6	F7
F1	1						
F2	–0.04	1					
F3	–0.19**	0.05*	1				
F4	0.16**	–0.12**	0.21**	1			
F5	–0.04	0.19**	–0.17**	–0.15**	1		
F6	0.06**	0.06**	0.08**	0.09**	0.09**	1	
F7	–0.10**	0.07**	–0.10**	–0.16**	0.33**	–0.03	1

Note. Bold indicates standardized factor loading >|0.30|. Italics denotes a reverse-coded item. BAS= Behavioral Activation Scale; UPPS= Urgency, Premeditation, Perseverance, Sensation-Seeking; EATQ= Early Adolescent Temperament Questionnaire; EF= Executive Function; BL= Baseline; Y1= Year 2 Follow-up; Y2= Year 2 Follow-up. F1-F4= Factors 1-4, respectively. Y_DD_A_2= Youth KSADS-5 depressive disorders at Year 2 follow-up; Y_AD_A_2= Youth KSADS-5 anxiety disorders at Year 2 follow-up; DD_A_2= Parent KSADS-5 depressive disorders at Year 2 follow-up; AD_A_2= Parent KSADS-5 depressive disorders at Y2 follow-up; CBCL_INT= Parent Child Behavior Checklist internalizing problems at Year 2 follow-up. BPMY_INT= Brief Problem Monitor – Youth internalizing problems at Year 2 follow-up; BPMT_INT= Brief Problem Monitor-Teacher internalizing problems at Year 2 follow-up; OCD_A_2= Parent KSADS-5 Obsessive Compulsive Disorder at Year 2 follow-up; Y_DSRD_A_2= Youth KSADS-5 disruptive disorders at Year 2 follow-up; ADHD_A_2= Parent KSADS -5 Attention Deficit and Hyperactivity Disorder at Year 2 follow-up ; DSRD_A_2= Parent KSADS-5 Disruptive Disorders at Year 2 follow-up ; CBCL_ATT= Parent Child Behavior Checklist attention problems at Year 2 follow-up; CBCL_EXT= Parent Child Behavior Checklist externalizing problems at Year 2 follow-up; BPMY_ATT= Brief Problem Monitor – Youth attention problems at Year 2 follow-up; BPMY_EXT= Brief Problem Monitor – Youth externalizing problems at Year 2 follow-up; BPMT_ATT= Brief Problem Monitor – Teacher attention problems at Year 2; BPMT_EXT= Brief Problem Monitor – Teacher externalizing problems at Year 2 follow-up; Y_BD_A_2= Youth KSADS-5 Bipolar Disorders at Year 2 follow-up; BD_A_2= Parent KSADS-5 Bipolar Disorders at Year 2 follow-up; CBCL_THT= Parent Child Behavior Checklist thought problems at Year 2 follow-up; CBCL_SOC= Parent Child Behavior Checklist social problems at Year 2 follow-up.

Table S13. 7-factor Final Model ESEM with Psychopathology Solution for Replication Arm 2

Item	Instrument Timepoint	F1	F2	F3	F4	F5	F6	F7	Internalizing Indicator	INT	Externalizing Indicator	EXT
I feel excited and full of energy when I get something that I want(bisbas8)	BAS BL	0.64	-0.07	0.10	0.07	-0.02	-0.07	0.02	Y_DD_A_2	0.42	Y_DSRD_A_2	0.34
When I am doing well at something, I like to keep doing this(bisbas9)	BAS BL	0.56	0.00	-0.17	-0.09	0.04	0.04	0.01	Y_AD_A_2	0.42	ADHD_A_2	0.69
I get thrilled when good things happen to me (bis bas 10)	BAS BL	0.69	0.02	-0.08	-0.05	0.02	-0.06	-0.02	DD_A_2	0.55	DSRD_A_2	0.56
It would excite me to win a contest(bisbas11)	BAS BL	0.62	0.01	-0.03	-0.10	0.01	0.01	-0.02	AD_A_2	0.43	CBCL_ATT	0.72
I get really excited when I see an opportunity to get something I like(bisbas12)	BAS BL	0.70	-0.05	0.05	-0.01	-0.01	-0.05	-0.01	CBCL_INT	0.65	CBCL_EXT	0.59
I often do things for no other reason than they might be fun(bisbas17)	BAS BL	0.35	-0.02	0.08	0.28	-0.01	0.13	0.00	BPMY_INT	0.39	BPMY_ATT	0.48
I am always willing to try something new, when I think it will be fun(bisbas19)	BAS BL	0.48	0.00	-0.15	0.04	-0.03	0.20	0.01	BPMT_INT	0.47	BPMY_EXT	0.37
I crave excitement and new sensations(bisbas18)	BAS BL	0.49	0.06	-0.09	0.09	0.01	0.17	-0.02	OCD_A_2	0.43	BPMT_ATT	0.57

Cash Choice	Cash Choice Task	0.09	-0.01	0.05	0.02	0.03	0.03	-0.04	BPMT_EXT	0.44
Opens presents before s/he is supposed to (eatq_open_present)	EATQ Y2	-0.02	0.20	0.02	-0.03	-0.02	-0.01	0.45	Y_BD_A_2	0.18
Would be frightened by the thought of skiing fast down a steep slope (eatq_ski_slope)	EATQ Y2	-0.01	-0.07	0.03	-0.03	0.01	0.53	0.27	BD_A_2	0.32
Usually does something fun for awhile before starting her/his homework, even though s/he is not supposed to (eatq_before_hw)	EATQ Y2	0.01	-0.06	0.02	-0.01	0.46	-0.05	0.16	CBCL_THT	0.42
Wouldn't be afraid to try a risky sport like deep sea diving (eatq_sea_dive)	EATQ Y2	0.00	-0.01	0.02	-0.06	0.08	0.62	0.04	CBCL_SOC	0.47
Would like driving a racing car (eatq_race_car)	EATQ Y2	0.01	-0.04	0.04	-0.04	0.02	0.56	-0.04		
Wouldn't want to go on the frightening rides at the fair (eatq_rides_scared)	EATQ Y2	0.03	-0.04	0.04	-0.04	0.03	0.46	0.20		
I enjoy taking risks (upps12)	UPPS BL	0.06	0.20	0.04	0.24	0.03	0.30	-0.06		
I like new, thrilling things, even if they	UPPS BL	0.16	0.17	-0.07	0.16	-0.04	0.34	-0.02		

are a little scary(upps18)								
I would like to learn to fly an airplane(upps21)	UPPS BL	-0.12	-0.05	-0.28	0.05	-0.05	0.30	-0.06
I would like to ski very fast down a high mountain slope (upps27)	UPPS BL	-0.03	0.10	-0.15	0.11	-0.04	0.47	-0.03
I often do things on the spur of the moment(bisbas20)	BAS BL	0.33	0.02	0.06	0.25	-0.03	0.14	0.02
Has a hard time finishing things on time (eatq_finish)	EATQ Y2	0.03	0.07	-0.01	0.03	0.61	0.00	0.33
Has a hard time waiting his/her turn to speak when excited (eatq_turn_taking)	EATQ Y2	-0.06	-0.04	-0.11	0.05	0.02	-0.03	0.54
When asked to do something does it right away, even if s/he doesn't want to (eatq_right_away)	EATQ Y2	-0.02	-0.05	-0.04	-0.01	0.49	0.02	0.10
Is more likely to do something s/he shouldn't do the more s/he tries to stop her/himself (eatq_impulse)	EATQ Y2	-0.02	0.15	-0.02	-0.04	0.05	0.06	0.51
Usually finishes her/his homework before it's due (eatq_finish_hw)	EATQ Y2	-0.02	0.06	0.01	-0.02	0.80	0.00	-0.14

Usually gets started right away on difficult assignments (eatq_early_start)	EATQ Y2	-0.03	-0.02	-0.01	0.00	0.94	0.02	-0.22
Usually puts off working on a project until it is due (eatq_puts_off)	EATQ Y2	0.01	-0.07	0.02	0.03	0.77	-0.03	0.03
Is able to stop him/herself from lauging at inappropriate times (eatq_laugh_control)	EATQ Y2	-0.08	0.13	-0.03	-0.05	0.06	0.09	0.07
Is often in the middle of doing one thing and then goes off to do something else without finishing it (eatq_sidetracked)	EATQ Y2	0.03	0.01	0.01	0.01	0.42	-0.04	0.40
Is usually able to stick with his/her plans and goals (eatq_stick_to_plan)	EATQ Y2	-0.05	0.11	-0.04	-0.02	0.51	0.06	0.09
I like to stop and think about things before I do it (upps6)	UPPS BL	-0.01	-0.03	0.71	0.03	0.00	-0.02	-0.02
When I feel bad, I often do things I later regret in order to make myself feel better now (upps7)	UPPS BL	0.02	-0.07	-0.14	0.36	0.00	-0.05	-0.01

I try to take a careful approach to things(upps16)	UPPS BL	-0.11	-0.05	0.55	-0.05	-0.02	0.11	0.02
When I am upset I often act without thinking(upps17)	UPPS BL	0.08	0.10	0.20	0.36	0.01	-0.04	-0.11
I tend to get things done on time(upps19)	UPPS BL	-0.08	-0.07	0.35	0.03	-0.30	-0.03	-0.01
I am very careful (upps23)	UPPS BL	-0.03	0.05	0.56	0.03	-0.06	0.15	-0.01
I tend to stop and think before doing things (upps28)	UPPS BL	0.01	0.07	0.78	0.05	0.01	0.01	-0.02
When I am in a great mood, I tend to do things that can cause me problems (upps35)	UPPS BL	-0.15	0.04	-0.01	0.72	0.01	0.00	0.00
I tend to act without thinking when I am very, very happy (upps36)	UPPS BL	0.03	-0.10	-0.04	0.71	0.00	-0.01	0.01
When I get really happy about something, I tend to do things that can lead to trouble (upps37)	UPPS BL	-0.09	-0.01	0.05	0.79	-0.01	0.02	0.01
I tend to lose control when I am in a great mood (upps39)	UPPS BL	0.03	-0.09	0.01	0.69	-0.01	-0.01	0.02
Game of Dice	Task Y2	-0.03	0.16	-0.01	-0.05	0.06	-0.01	0.01
Delay Discounting-ln(k)	Task Y1	0.02	-0.11	0.00	0.07	-0.01	-0.06	-0.01

Flanker	Task BL	0.02	0.57	0.02	0.01	-0.02	0.00	-0.01
List Sort	Task BL	-0.02	0.53	0.05	-0.05	0.05	-0.02	0.03
Card Sort	Task BL	-0.01	0.65	-0.02	-0.01	0.02	-0.02	-0.01
Nback	Task BL	-0.01	0.54	0.03	-0.01	0.00	0.00	0.07
SST	Task BL	0.00	-0.26	0.01	0.01	-0.01	0.07	-0.05

Factor Correlations	F1	F2	F3	F4	F5	F6	F7
F1	1						
F2	–0.03	1					
F3	–0.18**	–0.18**	1				
F4	0.19**	0.19**	0.19**	1			
F5	–0.05*	0.20**	–0.14**		1		
F6	0.07**	0.07**	0.05*	0.09**	0.05	1	
F7	–0.12**	0.08**	–0.07**	–0.17**	0.40**	–0.03	1

Note. Bold indicates standardized factor loading >|0.30|. Italics denotes a reverse-coded item. BAS= Behavioral Activation Scale; UPPS= Urgency, Premeditation, Perseverance, Sensation-Seeking; EATQ= Early Adolescent Temperament Questionnaire; EF= Executive Function; BL= Baseline; Y1= Year 2 Follow-up; Y2= Year 2 Follow-up. F1-F4= Factors 1-4, respectively. Y_DD_A_2= Youth KSADS-5 depressive disorders at Year 2 follow-up; Y_AD_A_2= Youth KSADS-5 anxiety disorders at Year 2 follow-up; DD_A_2= Parent KSADS-5 depressive disorders at Year 2 follow-up; AD_A_2= Parent KSADS-5 depressive disorders at Y2 follow-up; CBCL_INT= Parent Child Behavior Checklist internalizing problems at Year 2 follow-up. BPMY_INT= Brief Problem Monitor – Youth internalizing problems at Year 2 follow-up; BPMT_INT= Brief Problem Monitor-Teacher internalizing problems at Year 2 follow-up; OCD_A_2= Parent KSADS-5 Obsessive Compulsive Disorder at Year 2 follow-up; Y_DSRD_A_2= Youth KSADS-5 disruptive disorders at Year 2 follow-up; ADHD_A_2= Parent KSADS -5 Attention Deficit and Hyperactivity Disorder at Year 2 follow-up ; DSRD_A_2= Parent KSADS-5 Disruptive Disorders at Year 2 follow-up ; CBCL_ATT= Parent Child Behavior Checklist attention problems at Year 2 follow-up; CBCL_EXT= Parent Child Behavior Checklist externalizing problems at Year 2 follow-up; BPMY_ATT= Brief Problem Monitor – Youth attention problems at Year 2 follow-up; BPMY_EXT= Brief Problem Monitor – Youth externalizing problems at Year 2 follow-up; BPMT_ATT= Brief Problem Monitor – Teacher attention problems at Year 2; BPMT_EXT= Brief Problem Monitor – Teacher externalizing problems at Year 2 follow-up; Y_BD_A_2= Youth KSADS-5 Bipolar Disorders at Year 2 follow-up; BD_A_2= Parent KSADS-5 Bipolar Disorders at Year 2 follow-up; CBCL_THT= Parent

Child Behavior Checklist thought problems at Year 2 follow-up; CBCL_SOC= Parent Child Behavior Checklist social problems at Year 2 follow-up.

Table S14. Replication Arms Fit Statistics: ESEM Without Covariates, Psychopathology, or Rater Factors

	Fit Statistics (Replication Arm 1, Replication Arm 2)			
Number of Factors	χ^2 (df)	CFI	RMSEA	SRMR
1	63377.85 (1080), 61768.90 (1080)	0.468, 0.472	0.100, 0.099	0.115, 0.114
2	43272.97 (1033), 41660.57 (1033)	0.639, 0.646	0.084, 0.083	0.088, 0.086
3	26851.26 (987), 26631.01 (987)	0.779, 0.777	0.068, 0.067	0.062, 0.062
4	18405.51 (942), 18113.65 (1031)	0.851, 0.851	0.057, 0.056	0.050, 0.050
5	13056.48 (898), 12459.45 (898)	0.896, 0.899	0.049, 0.047	0.039, 0.039
6	9001.7 (855), 8246.02 (855)	0.930, 0.936	0.041, 0.039	0.032, 0.030
**7**	**6288.08 (813), 5850.82(813)**	**0.953, 0.956**	**0.033, 0.033**	**0.026, 0.025**
8	4479.71 (772), 4263.32 (772)	0.968, 0.970	0.029, 0.028	0.021, 0.020
9	3663.97 (732), 3477.46 (732)	0.975, 0.976	0.026, 0.026	0.019, 0.019
7-Factor ESEM with covariates and psychopathology factor outcomes	6164.18 (893), 6071.50 (893)	0.955, 0.955	0.032, 0.032	0.024, 0.024

Note: Boldface type indicates selected models. All $\chi2$ *p*<.001. $\chi2$= Chi-Square; CFI= Comparative Fit Index ; RMSEA= Root Mean Square Error of Approximation; SRMR= Standardized Root Mean Square Residual.

Table S15. Full Final 7-Factor ESEM with Psychopathology Factors and Rater Survey and Symptom Factors

Indicators	F1	F2	F3	F4	F5	F6	F7	P_SUR	Y_SUR	INT	EXT	Y_SYM	P_SYM	T_SYM
I feel excited and full of energy when I get something that I want (bisbas8)	**0.63**	-0.07	0.11	0.06	-0.02	-0.07	0.02		0.20					
When I am doing well at something, I like to keep doing this (bisbas9)	**0.56**	0.01	-0.16	-0.10	0.02	0.02	0.01		0.20					
I get thrilled when good things happen to me (bis bas 10)	**0.68**	0.01	-0.06	-0.05	0.03	-0.08	-0.02		0.20					
It would excite me to win a contest (bisbas11)	**0.61**	0.00	-0.03	-0.10	0.00	0.02	0.02		0.20					
I get really excited when I see an opportunity to get something I like (bisbas12)	**0.69**	-0.07	0.05	-0.02	-0.03	-0.04	0.01		0.20					
I often do things for no other reason than they might be fun (bisbas17)	**0.36**	-0.02	0.07	0.28	-0.02	0.11	-0.01		0.20					
I am always willing to try something new, when I think it will be fun (bisbas19)	**0.48**	-0.01	-0.16	0.05	-0.01	0.20	0.00		0.20					
I crave excitement and new sensations (bisbas18)	**0.50**	0.06	-0.10	0.09	0.01	0.18	-0.04		0.20					
Cash Choice	0.09	-0.02	0.05	0.01	0.03	0.03	-0.05		-0.20					

Opens presents before s/he is supposed to (eatq_open_present)	-0.02	0.20	0.01	-0.05	-0.03	0.02	**0.45**	-0.25	
Would be frightened by the thought of skiing fast down a steep slope (eatq_ski_slope)	-0.01	-0.07	0.03	-0.03	0.02	**0.51**	**0.31**	0.25	
Usually does something fun for awhile before starting her/his homework, even though s/he is not supposed to (eatq_before_hw)	0.00	-0.07	0.02	-0.01	**0.44**	-0.05	0.20	-0.25	
Wouldn't be afraid to try a risky sport like deep sea diving (eatq_sea_dive)	-0.01	-0.01	0.04	-0.05	0.11	**0.62**	0.04	0.25	
Would like driving a racing car (eatq_race_car)	0.01	-0.03	0.03	-0.04	0.01	**0.55**	-0.04	0.25	
Wouldn't want to go on the frightening rides at the fair (eatq_rides_scared)	0.04	-0.04	0.04	-0.03	0.04	**0.44**	0.23	0.25	
I enjoy taking risks (upps12)	0.08	0.18	0.05	0.21	0.01	**0.31**	-0.04		0.20
I like new, thrilling things, even if they are a little scary (upps18)	0.17	0.14	-0.07	0.15	-0.03	**0.34**	-0.02		0.20

I would like to learn to fly an airplane (upps21)	-0.10	-0.07	-0.29	0.03	-0.06	**0.30**	-0.03		0.20
I would like to ski very fast down a high mountain slope (upps27)	-0.01	0.08	-0.14	0.08	-0.05	**0.47**	-0.01		0.20
I often do things on the spur of the moment (bisbas20)	**0.36**	0.02	0.06	0.25	0.00	0.15	-0.02		0.20
Has a hard time finishing things on time (eatq_finish)	0.03	0.08	-0.01	0.03	**0.57**	-0.01	**0.35**	-0.25	
Has a hard time waiting his/her turn to speak when excited (eatq_turn_taking)	-0.07	-0.02	-0.12	0.05	0.01	-0.02	**0.55**	-0.25	
When asked to do something does it right away, even if s/he doesn't want to (eatq_right_away)	-0.03	-0.05	-0.06	0.01	**0.46**	0.03	0.14	-0.25	
Is more likely to do something s/he shouldn't do the more s/he tries to stop her/himself (eatq_impulse)	0.01	0.15	-0.01	-0.04	0.07	0.04	**0.50**	-0.25	
Usually finishes her/his homework before it's due (eatq_finish_hw)	-0.01	0.06	0.00	-0.03	**0.80**	0.00	-0.15	-0.25	
Usually gets started right away on	-0.02	-0.03	-0.01	0.00	**0.94**	0.02	-0.19	-0.24	

difficult assignments (eatq_early_start)									
Usually puts off working on a project until it is due (eatq_puts_off)	0.02	-0.08	0.02	0.02	**0.75**	-0.03	0.07	-0.25	
Is able to stop him/herself from lauging at inappropriate times (eatq_laugh_control)	-0.06	0.15	-0.03	-0.06	0.07	0.09	0.07	-0.25	
Is often in the middle of doing one thing and then goes off to do something else without finishing it (eatq_sidetracked)	0.03	0.02	0.02	0.01	**0.40**	-0.04	**0.42**	-0.25	
Is usually able to stick with his/her plans and goals (eatq_stick_to_plan)	-0.05	0.12	-0.04	-0.02	**0.50**	0.07	0.08	-0.25	
I like to stop and think about things before I do it (upps6)	0.00	-0.04	**0.71**	0.01	-0.01	-0.01	-0.02		0.20
When I feel bad, I often do things I later regret in order to make myself feel better now (upps7)	0.03	-0.07	-0.12	**0.36**	-0.01	-0.04	0.01		0.20
I try to take a careful approach to things (upps16)	-0.12	-0.03	**0.53**	-0.03	-0.04	0.12	0.03		0.20

When I am upset I often act without thinking (upps17)	0.08	0.11	0.20	**0.38**	0.01	-0.06	-0.09	0.20
I tend to get things done on time (upps19)	-0.09	-0.07	**0.36**	0.03	-0.27	-0.03	0.01	0.20
I am very careful (upps23)	-0.03	0.06	**0.57**	0.03	-0.04	0.13	-0.01	0.20
I tend to stop and think before doing things (upps28)	0.01	0.05	**0.80**	0.04	0.02	0.01	-0.01	0.20
When I am in a great mood, I tend to do things that can cause me problems (upps35)	-0.15	0.03	-0.01	**0.70**	0.01	0.02	-0.01	0.20
I tend to act without thinking when I am very, very happy (upps36)	0.02	-0.11	-0.04	**0.69**	0.00	-0.01	0.02	0.20
When I get really happy about something, I tend to do things that can lead to trouble (upps37)	-0.08	0.01	0.05	**0.79**	0.00	0.02	0.01	0.20
I tend to lose control when I am in a great mood (upps39)	0.02	-0.11	-0.01	**0.69**	0.00	-0.01	0.02	0.20
Game of Dice	-0.03	0.17	-0.01	-0.05	0.05	-0.02	-0.01	
Delay Discounting-ln(k)	0.02	-0.12	-0.01	0.08	-0.01	-0.06	0.02	
Flanker	0.01	**0.58**	0.02	0.01	-0.02	-0.01	0.00	
List Sort	-0.02	**0.54**	0.05	-0.04	0.04	0.00	0.02	

Card Sort	-0.01	**0.64**	-0.02	0.00	0.03	-0.03	0.00							
Nback	-0.02	**0.53**	0.01	0.00	0.00	-0.01	0.05							
SST	-0.01	-0.25	0.03	0.00	0.01	0.07	-0.05							
Y_DD_A_2										**0.45**		**0.55**		
Y_AD_A_2										**0.44**		**0.55**		
DD_A_2										**0.52**			**0.48**	
AD_A_2										**0.45**			**0.48**	
CBCL_INT										**0.66**			**0.48**	
BPMY_INT										**0.40**		**0.56**		
BPMT_INT										**0.47**				**0.48**
OCD_A_2										**0.42**			**0.48**	
Y_DSRD_A_2											**0.39**	**0.55**		
ADHD_A_2											**0.70**		**0.48**	
DSRD_A_2											**0.57**		**0.48**	
CBCL_ATT											**0.72**		**0.48**	
CBCL_EXT											**0.60**		**0.48**	
BPMY_ATT											**0.46**	**0.55**		
BPMY_EXT											**0.37**	**0.55**		
BPMT_ATT											**0.56**			**0.50**
BPMT_EXT											**0.45**			**0.49**
Y_BD_A_2											0.23	**0.55**		
BD_A_2											**0.37**		**0.48**	
CBCL_THT											**0.44**		**0.48**	
CBCL_SOC											**0.48**		**0.48**	
Factor Correlations	F1	F2	F3	F4	F5	F6	F7	P_SUR	Y_SUR	INT	EXT	Y_SYM	P_SYM	T_SYM
F1	1													
F2	-0.04**	1												
F3	-0.19**	0.05**	1											

F4	0.18**	-0.13**	0.20**	1										
F5	-0.05**	0.19**	-0.16**	-0.18**	1									
F6	0.06**	0.07**	0.07**	0.09**	0.06**	1								
F7	-0.11**	0.07**	-0.08**	-0.16**	0.41**	-0.02	1							
P_SUR	-	-	-	-	-	-	-	1						
Y_SUR	-	-	-	-	-	-	-	-	1					
INT	-	-	-	-	-	-	-	-	-	1				
EXT	-	-	-	-	-	-	-	-	-	0.52**	1			
Y_SYM	-	-	-	-	-	-	-	-	0.44**	-	-	1		
P_SYM	-	-	-	-	-	-	-	0.35**	-	-	-	-	1	
T_SYM	-	-	-	-	-	-	-	-	-	-	-	-	-	1

Note. Bold indicates standardized factor loading >|0.30|. Italics denotes a reverse-coded item. BAS= Behavioral Activation Scale; UPPS= Urgency, Premeditation, Perseverance, Sensation-Seeking; EATQ= Early Adolescent Temperament Questionnaire; EF= Executive Function; BL= Baseline; Y1= Year 2 Follow-up; Y2= Year 2 Follow-up. F1-F7= Factors 1-7, respectively. Y_DD_A_2= Youth KSADS-5 depressive disorders at Year 2 follow-up; Y_AD_A_2= Youth KSADS-5 anxiety disorders at Year 2 follow-up; DD_A_2= Parent KSADS-5 depressive disorders at Year 2 follow-up; AD_A_2= Parent KSADS-5 depressive disorders at Y2 follow-up; CBCL_INT= Parent Child Behavior Checklist internalizing problems at Year 2 follow-up. BPMY_INT= Brief Problem Monitor – Youth internalizing problems at Year 2 follow-up; BPMT_INT= Brief Problem Monitor-Teacher internalizing problems at Year 2 follow-up; OCD_A_2= Parent KSADS-5 Obsessive Compulsive Disorder at Year 2 follow-up; Y_DSRD_A_2= Youth KSADS-5 disruptive disorders at Year 2 follow-up; ADHD_A_2= Parent KSADS -5 Attention Deficit and Hyperactivity Disorder at Year 2 follow-up ; DSRD_A_2= Parent KSADS-5 Disruptive Disorders at Year 2 follow-up ; CBCL_ATT= Parent Child Behavior Checklist attention problems at Year 2 follow-up; CBCL_EXT= Parent Child Behavior Checklist externalizing problems at Year 2 follow-up; BPMY_ATT= Brief Problem Monitor – Youth attention problems at Year 2 follow-up; BPMY_EXT= Brief Problem Monitor – Youth externalizing problems at Year 2 follow-up; BPMT_ATT= Brief Problem Monitor – Teacher attention problems at Year 2; BPMT_EXT= Brief Problem Monitor – Teacher externalizing problems at Year 2 follow-up; Y_BD_A_2= Youth KSADS-5 Bipolar Disorders at Year 2 follow-up; BD_A_2= Parent KSADS-5 Bipolar Disorders at Year 2 follow-up; CBCL_THT= Parent Child Behavior Checklist thought problems at Year 2 follow-up; CBCL_SOC= Parent Child Behavior Checklist social problems at Year 2 follow-up; P_SUR= Parent survey/ESEM rater factor; Y_SUR= Youth survey/ESEM rater factor; INT= Internalizing psychopathology factor; EXT= Externalizing psychopathology factor; Y_SYM= Youth symptom/psychopathology rater factor; P_SYM= Parent symptom/psychopathology rater factor; T_SYM= Teacher symptom/psychopathology rater factor.

Table S16. Loadings for 2-Factor with Psychopathology CFA and CFA Rater Factors

Indicators	Factor Loadings				
	Factor 1 (INT)	Factor 2 (EXT)	Youth Symptom Factor	Parent Symptom Factor	Teacher Symptom Factor
Youth KSADS Depressive Disorders	**0.50**		**0.52**		
Youth KSADS Anxiety Disorders	**0.54**		**0.52**		
Youth BPM Internalizing	**0.47**		**0.53**		
Parent KSADS Depressive Disorders	**0.44**			**0.51**	
Parent KSADS Anxiety Disorders	**0.40**			**0.51**	
Parent CBCL Internalizing	**0.61**			**0.51**	
Teacher BPM Internalizing	**0.46**			**0.51**	**0.48**
Youth KSADS Disruptive Disorders		**0.44**	**0.52**		
Youth BPM Attention Problems		**0.49**	**0.52**		
Youth BPM Externalizing		**0.43**	**0.52**		
Parent KSADS Disruptive Disorders		**0.53**		**0.51**	
Parent KSADS ADHD		**0.58**		**0.51**	
Parent CBCL Attention Problems		**0.63**		**0.52**	
Parent CBCL Externalizing		**0.60**		**0.51**	
Teacher BPM Attention Problems		**0.55**			**0.50**
Teacher BPM Externalizing		**0.50**			**0.48**
Youth KSADS Bipolar Disorders		0.27	**0.52**		
Parent KSADS Bipolar Disorders		**0.33**		**0.51**	
Parent KSADS Obsessive Compulsive Disorder	**0.36**			**0.51**	
Parent CBCL Social Problems		**0.46**		**0.51**	
Parent CBCL Thought Problems		**0.50**		**0.51**	

Note. Bold indicates standardized factor loading >|0.30|. KSADS= Kiddie Schedule of Affective Disorders and Schizophrenia; CBCL= Childhood Behavior Checklist; BPM= Brief Problem Monitor.

Table S17. Correlations Between Psychopathology Measures

	1	2	3	4	5	6	7	8	9	10	11	12	13	14	15	16	17	18	19	20	21
1 Y DEP Y2	1.00																				
2 Y BIP Y2	0.31	1.00																			
3 Y ANX Y2	0.54	0.45	1.00																		
4 Y DSRD Y2	0.36	0.41	0.24	1.00																	
5 P DEP Y2	0.35	0.14	0.30	0.25	1.00																
6 P BIP Y2	0.23	0.22	0.11	0.21	0.08	1.00															
7 P ANX Y2	0.23	0.07	0.32	0.07	0.49	0.36	1.00														
8 P OCD Y2	0.20	0.10	0.23	0.15	0.37	0.38	0.51	1.00													
9 P ADHD Y2	0.21	0.17	0.13	0.19	0.38	0.42	0.36	0.38	1.00												
10 P DSRD Y2	0.14	0.16	0.08	0.31	0.43	0.46	0.39	0.38	0.65	1.00											
11 P SCL Y2	0.20	0.15	0.17	0.19	0.37	0.38	0.35	0.33	0.46	0.45	1.00										
12 P THT Y2	0.18	0.12	0.20	0.16	0.35	0.41	0.39	0.45	0.46	0.41	0.55	1.00									
13 P ATT Y2	0.19	0.15	0.15	0.24	0.35	0.44	0.31	0.34	0.73	0.51	0.58	0.57	1.00								
14 P INT Y2	0.24	0.11	0.30	0.13	0.49	0.37	0.56	0.45	0.39	0.42	0.61	0.60	0.53	1.00							
15 P EXT Y2	0.19	0.17	0.13	0.33	0.39	0.45	0.29	0.31	0.51	0.67	0.62	0.55	0.63	0.58	1.00						
16 Y BPM INT Y2	0.47	0.36	0.59	0.24	0.23	0.13	0.22	0.18	0.12	0.07	0.18	0.17	0.14	0.25	0.12	1.00					
17 Y BPM ATT Y2	0.38	0.36	0.39	0.44	0.22	0.16	0.11	0.15	0.36	0.21	0.21	0.20	0.34	0.16	0.22	0.48	1.00				
18 Y BPM EXT Y2	0.34	0.33	0.25	0.57	0.18	0.19	0.08	0.11	0.25	0.30	0.19	0.17	0.22	0.15	0.31	0.41	0.52	1.00			
19 T BPM ATT Y2	0.15	0.17	0.05	0.28	0.17	0.20	0.08	0.12	0.45	0.28	0.24	0.17	0.41	0.09	0.29	0.11	0.31	0.20	1.00		
20 T BPM EXT Y2	0.14	0.16	0.05	0.22	0.15	0.22	0.06	0.10	0.32	0.32	0.25	0.16	0.29	0.11	0.34	0.10	0.22	0.25	0.57	1.00	
21 T BPM INT Y2	0.20	0.18	0.19	0.08	0.22	0.12	0.19	0.20	0.21	0.18	0.21	0.15	0.21	0.21	0.14	0.18	0.17	0.11	0.38	0.33	1.00

Note. Y_DEP_Y2= youth-reported depressive disorders at year 2; Y_BIP_Y2= youth-reported bipolar disorders at year2; Y_ANX_Y2= youth-reported anxiety disorders at year 2; Y_DIS_Y2= youth-reported disruptive disorders at year 2; P_DEP_Y2= parent-reported depressive disorders at year 2; P_BIP_Y2= parent-reported bipolar disorders at year 2; P_ANX_Y2= parent-reported anxiety disorders at year 2; ; P_OCD_Y2= parent-reported obsessive compulsive disorder at year 2; P_ADHD_Y2= parent-reported attention deficit and hyperactivity disorder at year 2; P_DIS_Y2= parent-reported disruptive disorders at year 2; SOCIAL_Y2= CBCL social problems at year 2; THOUGHT_Y2= CBCL thought problems at year 2; ATT_Y2= CBCL attention problems at year 2; INT_Y2= CBCL internalizing disorders at year 2; EXT_Y2= CBCL externalizing disorders at year 2. BPMY_INT_Y2= BPM youth-

reported internalizing summary scores measured at year 2 follow-up; BPMY_EXT_Y2= BPM youth reported externalizing problems summary score at year 2 follow-up; BPMY_ATT_Y2= BPM youth reported attention problems summary score at year 2 follow-up; BPMT_ATT_Y2= BPM teacher reported attention problems summary score at year 2 follow-up; BPMT_EXT_Y2= BPM teacher reported externalizing problems summary score at year 2 follow-up; BPMT_INT_Y2= BPM teacher-reported internalizing summary scores measured at year 2 follow-up.

Table S18. ESEM Factor Correlations with General CBCL Internalizing Summary Score Versus CBCL Withdraw/Depressed and Anxious/Depressed Subscale Scores

	ESEM Factors						
	BAS	PU	SS	EF	PRE	ACT	INH
CBCL Internalizing	-0.04*	-0.02*	-0.15**	0.09**	-0.00	-0.11**	-0.37**
CBCL withdrawn/depressed	-0.05**	0.00	-0.16**	0.02	0.00	-0.15**	-0.26**
CBCL anxious/depressed	-0.03*	-0.03*	-0.17**	0.10**	0.00	-0.05**	-0.35**

Note. ESEM= Exploratory Structural Equation Modelling; BAS= Behavioral Activation Scale; PU= Positive Urgency; SS= Sensation Seeking; EF= Executive Function; PRE= Lack of Premeditation; ACT= Activation Control; INH= Inhibitory Control; CBCL= Child Behavior Checklist. Values in parentheses indicate standard errors. *denotes p-value <0.05; **denotes p-value <0.001.

Table S19. Confirmatory Factor Analysis With Items Loading on Subscale Factors: Factor Loadings

Indicators	BAS RR	BAS FS	PU	NU	PRE	SS	HIP	ACT	INH	EF	RW
I feel excited and full of energy when I get something that I want (bisbas8)	0.641										
When I am doing well at something, I like to keep doing this (bisbas9)	0.586										
I get thrilled when good things happen to me (bis bas 10)	0.688										
It would excite me to win a contest (bisbas11)	0.619										
I get really excited when I see an opportunity to get something I like (bisbas12)	0.713										
I often do things for no other reason than they might be fun (bisbas17)		0.592									
I am always willing to try something new, when I think it will be fun (bisbas19)		0.591									
I crave excitement and new sensations (bisbas18)		0.624									
I often do things on the spur of the moment (bisbas20)		0.567									
When I am in a great mood, I tend to do things that can cause me problems (upps35)			0.661								
I tend to act without thinking when I am very, very happy (upps36)			0.721								
When I get really happy about something, I tend to do things that can lead to trouble (upps37)			0.799								

I tend to lose control when I am in a great mood (upps39)	0.725				
When I feel bad, I often do things I later regret in order to make myself feel better now (upps7)		0.387			
When I am upset I often act without thinking (upps17)		0.595			
I like to stop and think about things before I do it (upps6)			0.734		
I try to take a careful approach to things (upps16)			0.567		
I am very careful (upps23)			0.624		
I tend to stop and think before doing things (upps28)			0.803		
I enjoy taking risks (upps12)				0.547	
I like new, thrilling things, even if they are a little scary (upps18)				0.582	
I would like to learn to fly an airplane (upps21)				0.202	
I would like to ski very fast down a high mountain slope (upps27)				0.469	
Would be frightened by the thought of skiing fast down a steep slope (eatq_ski_slope)					0.645
Wouldn't be afraid to try a risky sport like deep sea diving (eatq_sea_dive)					0.642
Would like driving a racing car (eatq_race_car)					0.442
Wouldn't want to go on the frightening rides at the fair (eatq_rides_scared)					0.568

Usually does something fun for awhile before starting her/his homework, even though s/he is not supposed to (eatq_before_hw)	0.554	
Has a hard time finishing things on time (eatq_finish)	0.800	
When asked to do something does it right away, even if s/he doesn't want to (eatq_right_away)	0.345	
Usually finishes her/his homework before it's due (eatq_finish_hw)	0.727	
Usually gets started right away on difficult assignments (eatq_early_start)	0.801	
Usually puts off working on a project until it is due (eatq_puts_off)	0.759	
Opens presents before s/he is supposed to (eatq_open_present)		0.416
Has a hard time waiting his/her turn to speak when excited (eatq_turn_taking)		0.452
Is more likely to do something s/he shouldn't do the more s/he tries to stop her/himself (eatq_impulse)		0.525
Is able to stop him/herself from lauging at inappropriate times (eatq_laugh_control)		0.221
Is usually able to stick with his/her plans and goals (eatq_stick_to_plan)		0.637

Flanker										0.536	
List Sort										0.587	
Card Sort										0.649	
Nback										0.559	
SST										-0.281	
Game of Dice											0.205
Delay Discounting- ln(k)											-0.138
Cash Choice											-0.064
Factor Correlations	BAS RR	BAS FS	PU	NU	PRE	SS	HIP	ACT	INH	EF	RW
BAS RR	1										
BAS FS	0.67*	1									
PU	0.11*	0.36*	1								
NU	0.21*	0.34*	0.73*	1							
PRE	-0.25*	-0.06*	0.24*	0.29*	1						
SS	0.18*	0.55*	0.29*	0.23*	0.07*	1					
HIP	0.02	0.13*	0.02	-0.04	0.09*	0.49*	1				
ACT	-0.04*	-0.09*	-0.18*	-0.18*	-0.18*	-0.04	0.20*	1			
INH	-0.14*	-0.17*	-0.24*	-0.25*	-0.20*	-0.05	0.30*	0.80*	1		
EF	-0.08*	-0.09*	-0.19*	-0.07*	0.06*	0.12*	0.07*	0.22*	0.32*	1	
RW	-0.30*	-0.30*	-0.53	-0.35*	0.00	0.15	0.05	0.33*	0.53*		1

Note. Psychopathology and methods factor loadings not shown, but were included in the model. BAS RR= Behavioral Activation Scale reward responsiveness CFA factor. BAS FS= Behavioral Activation Scale fun-seeking CFA factor. PU= Urgency,

Perseverance, Premeditation, and Sensation Seeking Scale positive urgency CFA factor. NU= Urgency, Perseverance, Premeditation, and Sensation Seeking Scale negative urgency CFA factor. SS= Urgency, Perseverance, Premeditation, and Sensation Seeking Scale sensation seeking CFA factor. HIP= Early Adolescent Temperament Questionnaire high intensity pleasure CFA factor. PRE= Urgency, Perseverance, Premeditation, and Sensation Seeking Scale lack of premeditation CFA factor. ACT= Early Adolescent Temperament Questionnaire activation control CFA factor. INH= Early Adolescent Temperament Questionnaire inhibitory control CFA factor. EF= task-based executive function CFA factor. RW= task-based reward CFA factor. *Denotes a significant correlation at $p<0.01$.

Table S20. Confirmatory Factor Analysis With Items Loading Onto Subscale Factors: Relations to Psychopathology Factors

	Multiple Regression Model				Correlational Model			
**CFA Factor Predictor**	β to Internalizing	SE	β to Externalizing	SE	*r* with Internalizing	SE	*r* with Externalizing	SE
**BAS Reward Responsiveness**	0.002	0.085	-0.077	0.036	-0.013	0.017	0.102*	0.015
**BAS Fun-Seeking**	0.073	0.108	0.066	0.044	-0.031	0.018	0.153*	0.016
**Positive Urgency**	0.129	0.104	-0.164*	0.043	0.076*	0.016	0.233*	0.014
**Negative Urgency**	0.192	0.110	0.247*	0.047	0.179*	0.022	0.342*	0.021
**Sensation Seeking**	-0.244	0.111	-0.105	0.056	-0.114*	0.020	0.050*	0.018
**High Intensity Pleasure**	-0.136	0.085	0.148*	0.056	-0.407*	0.019	-0.184*	0.018
**Lack of Premeditation**	-0.062	0.050	0.022	0.026	0.079*	0.017	0.271*	0.014
**Activation Control**	-0.074	0.086	-0.089	0.042	-0.402*	0.015	-0.778*	0.011
**Inhibitory Control**	-0.483*	0.121	-0.889*	0.073	-0.440*	0.019	-0.927*	0.016
**EF**	-0.391*	0.092	0.042	0.037	-0.085*	0.017	-0.251*	0.015
**Reward Tasks**	0.682*	0.129	0.003	0.053	-0.171	0.075	-0.456*	0.115

Note: In this model the factors are the measure subscales. Items were allowed to load onto only one factor (see Supplemental Table S19). Items that did not originate from these subscales were loaded onto the factor that they had loaded onto most strongly in the exploratory analysis. *Denotes p<0.01

Table S21. Hierarchal CFA With Items Loading on Subscales and Subscales Loading on Instrument Factors

	**Multiple Regression Model**				**Correlational Model**			
**CFA Factor Predictor**	β to Internalizing	SE	β to Externalizing	SE	*r* with Internalizing	SE	*r* with Externalizing	SE
**BAS (**Reward Responsivess and Fun-Seeking Subscales**)**	0.008	0.044	0.010	0.012	-0.023	0.014	0.121*	0.013
**UPPS (**Positive, Negative Urgency, Sensation Seeking, Lack of Premeditation Subscales**)**	0.157	0.069	0.076*	0.019	0.089*	0.017	0.316*	0.015
**EATQ (**High Intensity Pleasure, Activation Control, Inhibitory Control Subscales**)**	-0.698*	0.055	-0.962*	0.016	-0.545*	0.015	-0.963*	0.011
**EF Tasks**	-0.596*	0.118	-0.025	0.033	-0.077*	0.017	-0.239*	0.015
**Reward Tasks**	0.831*	0.139	0.058	0.039	-0.106	0.051	-0.328*	0.064

Note: In this model the factors are the scales with items loading onto the CFA subscales as suggested in the parentheses and described above, but with these subscales now loading onto hierarchal scale factors. Items were allowed to load onto only one factor (see Supplemental Table S19). Items that did not originate from these subscales were loaded onto the factor that they had loaded onto most strongly in the exploratory analysis. *Denotes p<0.01

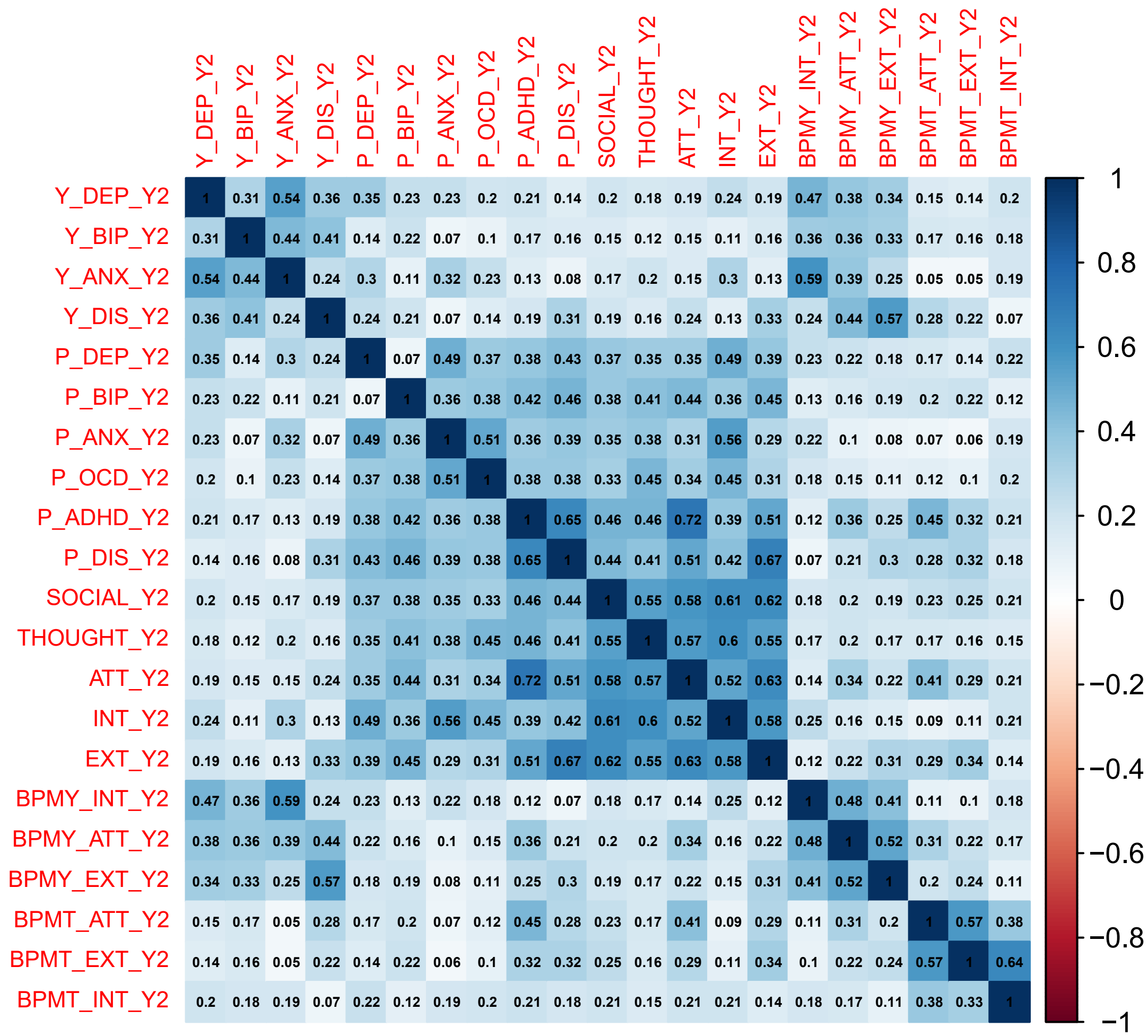

Figure S1. Correlation Matrix of Year 2 Psychopathology Measures. Y_DEP_Y2= youth-reported depressive disorders at year 2; Y_ANX_Y2= youth-reported anxiety disorders at year 2; Y_DIS_Y2= youth-reported disruptive disorders at year 2; P_DEP_Y2=

parent-reported depressive disorders at year 2; P_BIP_Y2= parent-reported bipolar disorders at year 2; P_ANX_Y2= parent-reported anxiety disorders at year 2; P_DIS_Y2= parent-reported disruptive disorders at year 2; P_OCD_Y2= parent-reported obsessive compulsive disorder at year 2; P_ADHD_Y2= parent-reported ADHD at year2; SOCIAL_Y2= CBCL social problems at year 2; THOUGHT_Y2= CBCL thought problems at year 2; ATT_Y2= CBCL attention problems at year 2; INT_Y2= CBCL internalizing disorders at year 2; EXT_Y2= CBCL externalizing disorders at year 2; BPMY_INT_Y2= BPM youth-reported internalizing summary scores measured at year 2 follow-up; BPMY_EXT_Y2= BPM youth reported externalizing problems summary score at year 2 follow-up; BPMY_ATT_Y2= BPM youth reported attention problems summary score at year 2 follow-up; BPMT_INT_Y2= BPM teacher-reported internalizing summary scores measured at year 2 follow-up; BPMT_EXT_Y2= BPM teacher reported externalizing problems summary score at year 2 follow-up; BPMT_ATT_Y2= BPM teacher reported attention problems summary score at year 2 follow-up.

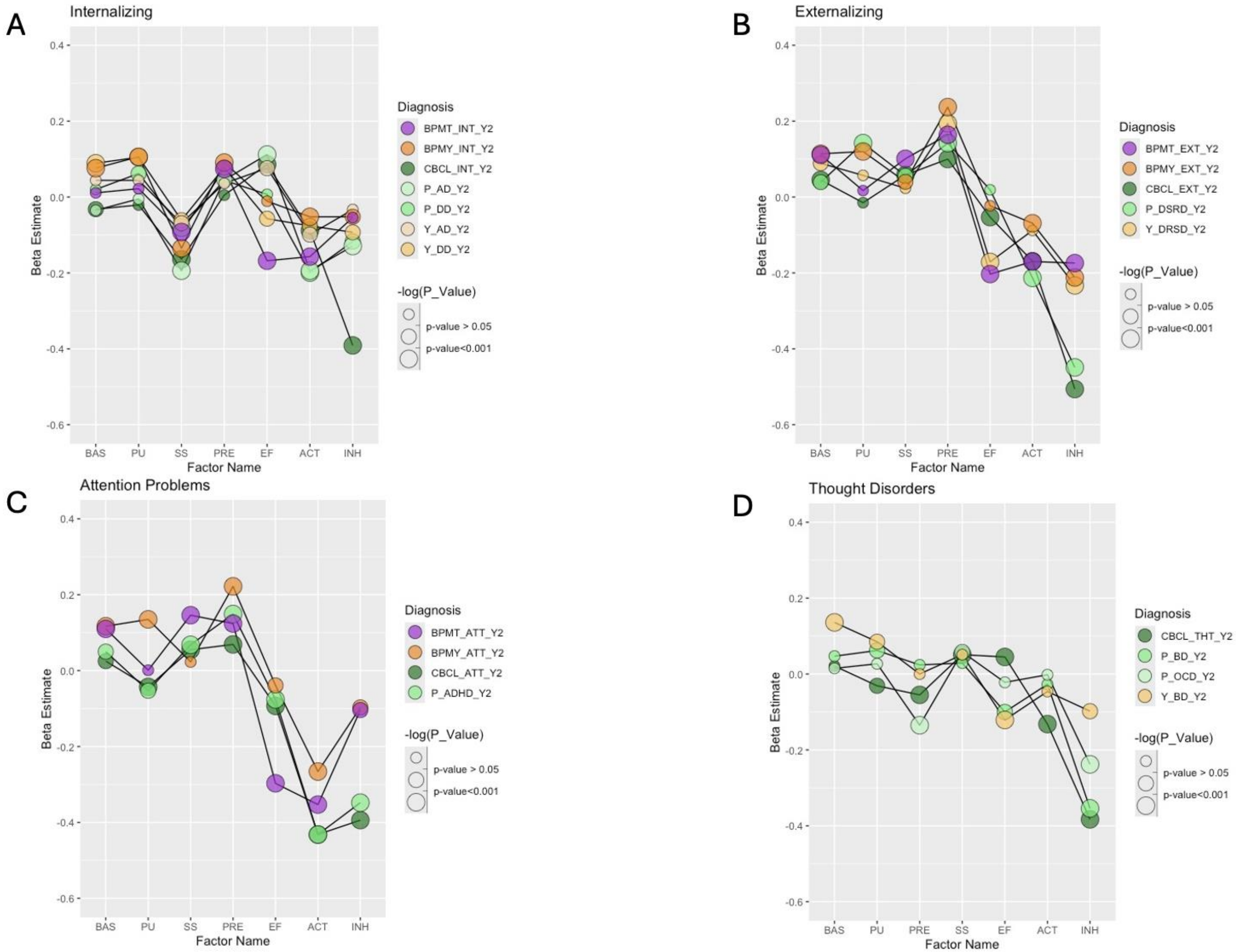

Figure S2. Standardized regression coefficient estimates between seven-factor model and externalizing/internalizing disorder diagnoses. BAS= BAS factor (factor 1), PU= Positive Urgency (factor 2), PRE= UPPS-P Lack of Premeditation factor (factor 3), SS= Sensation Seeking factor (factor 4), EF= Executive Function factor (factor 5), ACT= EATQ activation control factor (factor 6), INH= EATQ inhibitory control factor (factor 7). **A:** BPMT_INT_Y2= BPM teacher-reported internalizing summary scores measured at year

2 follow-up. BPMY_INT_Y2= BPM youth-reported internalizing summary scores measured at year 2 follow-up. CBCL_INT_Y2= CBCL internalizing summary scores measured at year 2 follow-up; P_AD_Y2= parent-reported KSADS-5 anxiety disorders diagnosis reported at year 2 follow-up; P_DD_Y2= parent-reported KSADS-5 depressive disorders diagnosis reported at year 2 follow-up; Y_AD_Y2= youth-reported KSADS-5 anxiety disorders diagnosis reported at year 2 follow-up; Y_DD_Y2= youth-reported KSADS-5 depressive disorders diagnosis reported at year 2 follow-up. **B:** BPMT_EXT_Y2= BPM teacher reported externalizing problems summary score at year 2 follow-up; BPMY_EXT_Y2= BPM youth reported externalizing problems summary score at year 2 follow-up; CBCL_EXT_Y2= CBCL externalizing summary score at year 2 follow-up; P_DSRD_Y2= parent/guardian reported KSADS-5 disruptive disorders at year 2 follow-up; Y_DSRD_Y2= youth reported KSADS-5 disruptive disorders at year 2 follow-up. **C**: BPMT_ATT_Y2= BPM teacher reported attention problems summary score at year 2 follow-up, BPMY_ATT_Y2= BPM youth reported attention problems summary score at year 2 follow-up, CBCL_ATT_Y2= CBCL attention problems summary score at year 2 follow-up; P_ADHD_Y2= Parent-reported KSADS-5 ADHD disorder at year 2 follow-up. **D**: CBCL_THT_Y2= CBCL thought problems at year 2; P_BD_Y2= Parent-reported KSADS-5 bipolar disorders diagnosis at year 2 follow-up; P_OCD_Y2= Parent-reported KSADS-5 OCD at year 2; Y_BD_Y2= youth-reported KSADS-5 bipolar disorder diagnoses at year 2 follow-up.
